# Early EULAR Target Achievement and Kidney Outcomes in Lupus Nephritis

**DOI:** 10.1016/j.ekir.2026.107079

**Published:** 2026-09-17

**Authors:** Ryosuke Umeda, Shun Minatoguchi, Hidekazu Ikeuchi, Keiju Hiromura, Naotake Tsuboi, Yukio Yuzawa, Yutaka Kawahito, Takahiro Seno, Kazuro Kamada, Michihito Kono, Akiho Iwashita, Akira Onishi, Kentaro Minowa, Naoto Tamura, Shinya Kaname, Takahisa Kawakami, Yoshiya Tanaka, Satoshi Kubo, Hayato Shimizu, Shuji Sumitomo, Kazuoto Hiramoto, Yuko Kaneko, Tomohiro Koga, Takuya Tomokawa, Hiroko Sato, Tomonori Ishii, Nobuyuki Yajima, Hirofumi Amano, Takayuki Kon, Shoichi Maruyama, Sawako Kato, Hiromi Shimada, Hiroaki Dobashi, Masahiro Ayano, Hiroaki Niiro, Shinsuke Yasuda, Tadashi Hosoya, Isao Matsumoto, Yuya Kondo, Yasunori Iwata, Shiori Nakagawa, Takahisa Gono, Masataka Kuwana, Tatsuya Atsumi, Koichiro Ohmura, Hiroki Hayashi

**Affiliations:** 1 Department of Nephrology, Fujita Health University School of Medicine, Toyoake, Japan; 2 Department of Nephrology and Rheumatology, Graduate School of Medicine, Gunma University, Maebashi, Japan; 3 Inflammation and Immunology, Kyoto Prefectural University of Medicine, Kyoto, Japan; 4 Department of Rheumatology, Endocrinology and Nephrology, Faculty of Medicine and Graduate School of Medicine, Hokkaido University, Sapporo, Japan; 5 Department of Rheumatology and Clinical Immunology, Graduate School of Medicine, Kyoto University, Kyoto, Japan; 6 Department of Advanced Medicine for Rheumatic Diseases, Graduate School of Medicine, Kyoto University, Kyoto, Japan; 7 Department of Internal Medicine and Rheumatology, Juntendo University School of Medicine, Tokyo, Japan; 8 Department of Nephrology and Rheumatology, Kyorin University School of Medicine, Mitaka, Japan; 9 Department of Internal Medicine, Kichijoji Asahi Hospital, Musashino, Japan; 10 The First Department of Internal Medicine, University of Occupational and Environmental Health, Kitakyushu, Japan; 11 Department of Molecular Targeted Therapeutics, University of Occupational and Environmental Health, Kitakyushu, Japan; 12 Department of General Internal Medicine, Kobe City Medical Center General Hospital, Kobe, Japan; 13 Department of Rheumatology, Kobe City Medical Center General Hospital, Kobe, Japan; 14 Division of Rheumatology, Department of Internal Medicine, Keio University School of Medicine, Tokyo, Japan; 15 Department of Immunology and Rheumatology, Nagasaki University Graduate School of Biomedical Sciences, Nagasaki, Japan; 16 Department of Rheumatology, Tohoku University Hospital, Sendai, Japan; 17 Department of Hematology and Rheumatology, Tohoku Medical and Pharmaceutical University, Sendai, Japan; 18 Division of Rheumatology, Department of Medicine, School of Medicine, Showa Medical University, Tokyo, Japan; 19 Department of Internal Medicine and Rheumatology, Juntendo University Nerima Hospital, Tokyo, Japan; 20 Department of Nephrology, Nagoya University Graduate School of Medicine, Nagoya, Japan; 21 Division of Hematology, Rheumatology and Respiratory Medicine, Department of Internal Medicine, Faculty of Medicine, Kagawa University, Miki-cho, Japan; 22 Department of Medicine and Biosystemic Science, Graduate School of Medical Sciences, Kyushu University, Fukuoka, Japan; 23 Department of Medical Education, Graduate School of Medical Sciences, Kyushu University, Fukuoka, Japan; 24 Department of Rheumatology, Institute of Science Tokyo, Tokyo, Japan; 25 Department of Rheumatology, Institute of Medicine, University of Tsukuba, Tsukuba, Japan; 26 Department of Nephrology and Rheumatology, Kanazawa University, Kanazawa, Japan; 27 Department of Allergy and Rheumatology, Graduate School of Medicine, Nippon Medical School, Tokyo, Japan; 28 Scleroderma/Myositis Center of Excellence, Nippon Medical School Hospital, Tokyo, Japan

**Keywords:** EULAR treatment targets, lupus nephritis, proteinuria

## Abstract

**Introduction:**

The prognostic value of achieving the European Alliance of Associations for Rheumatology (EULAR) 3- and 6-month treatment targets in lupus nephritis (LN) remains uncertain.

**Methods:**

We conducted a multicenter retrospective cohort study of adults with biopsy-proven proliferative LN. Patients were classified by EULAR target achievement: a ≥ 25% reduction in urine protein-to-creatinine ratio (UPCR) with preserved kidney function at 3 months and a ≥ 50% reduction in UPCR at 6 months. The primary outcome was a ≥ 30% decline in estimated glomerular filtration rate (eGFR) at 24 months from the higher of baseline or 3-month eGFR. Multivariable logistic regression and linear mixed-effects models were used.

**Results:**

Among 449 screened patients, 293 and 288 constituted the 3- and 6-month analytic cohorts, respectively. For the 3-month EULAR target analysis, the primary outcome occurred in 24/213 (11.3%) achievers and 18/80 (22.5%) nonachievers (adjusted odds ratio [OR], 0.40; 95% confidence interval [CI], 0.20–0.82). For the 6-month EULAR target analysis, the corresponding proportions were 31/246 (12.6%) and 11/42 (26.2%) (adjusted OR, 0.40; 95% CI, 0.17–0.91). Adjusted eGFR trajectories were higher in achievers, although separation preceded 6-month classification. In a 3-month landmark analysis, the composite target showed no clear incremental association beyond kidney function at 3 months, whereas the UPCR-reduction criterion remained associated with subsequent eGFR decline (OR, 0.34; 95% CI, 0.14–0.86).

**Conclusion:**

Achievement of the EULAR early treatment targets at 3 and 6 months was associated with better kidney outcomes in proliferative LN. Achievement by 3 months may identify patients with a particularly favorable renal prognosis.

## Graphical abstract

**Figure:**
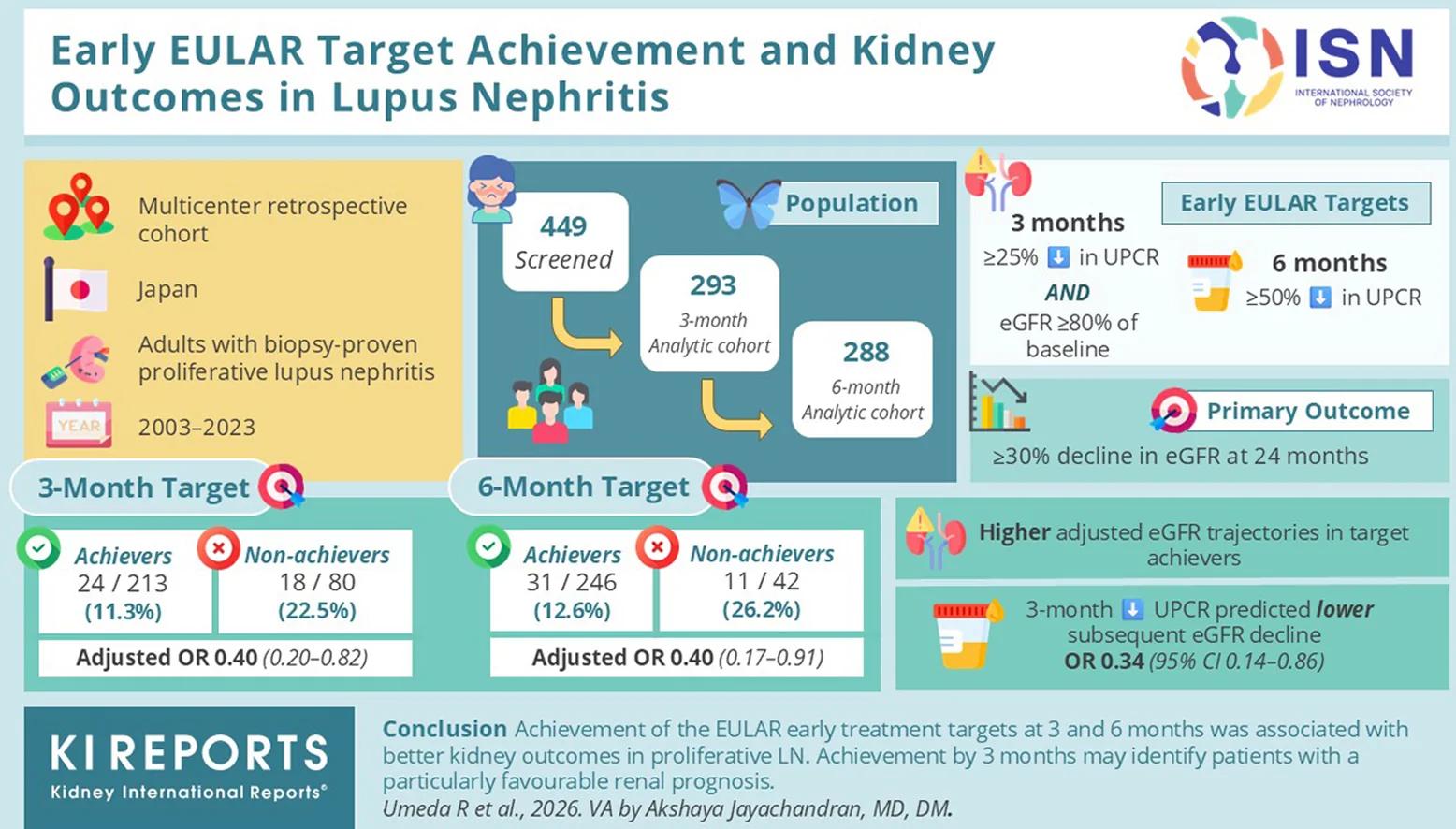

LN is one of the major organ manifestations in systemic lupus erythematosus (SLE) and an important cause of chronic kidney disease (CKD) and end-stage kidney disease in patients with SLE, contributing substantially to long-term morbidity and mortality.^1^ The overarching goal of treatment in LN is to preserve kidney function and prevent irreversible renal damage, consistent with international guidelines.^2^^,^^3^

Because long-term kidney outcomes require prolonged follow-up, surrogate markers are widely used to assess treatment response. Among these, reduction in proteinuria, typically measured by the UPCR, has been shown to correlate with renal prognosis and is commonly used to define treatment response. Several studies have suggested that achieving remission, defined as reduction in proteinuria below a given threshold, with or without stable or improved serum creatinine or eGFR, particularly by 12 months, is associated with a lower risk of subsequent renal function decline and progression to end-stage kidney disease.^4^, ^5^, ^6^, ^7^, ^8^, ^9^ To provide a standardized framework for response evaluation, the EULAR and the European Renal Association–European Dialysis and Transplant Association jointly proposed target-based treatment goals in 2019 and updated them in 2025, recommending a ≥ 25% reduction in UPCR by 3 months with stable or improved kidney function, a ≥ 50% reduction by 6 months, and a UPCR < 0.7 g/g by 12 months.^10^^,^^11^ These targets are increasingly adopted as early indicators of treatment response; however, the extent to which early EULAR target achievement is associated with subsequent kidney function decline remains incompletely defined, particularly for the 3-month target.

For the 6-month time point, several studies have suggested that failure to achieve early proteinuria or remission milestones is associated with poor long-term renal outcomes, including end-stage kidney disease and persistent renal dysfunction.^12^, ^13^, ^14^, ^15^ However, these studies used heterogeneous response definitions, and only a limited number evaluated a response definition closely aligned with the 6-month target proposed in the EULAR recommendations. Evidence for the 3-month target is more limited and less direct, with available studies often assessing whether early proteinuria changes predict later renal response or short-term treatment response, which are themselves surrogate outcomes, rather than subsequent decline in kidney function itself.^16^^,^^17^

To address this gap, we conducted a retrospective cohort study of patients with proliferative LN to examine whether achieving the EULAR-recommended treatment targets at 3 and 6 months was associated with a clinically meaningful short-term kidney outcome, defined as a ≥ 30% decline in eGFR at 24 months.

## Methods

### Study Design and Population

This study was a multicenter, retrospective cohort analysis conducted within the multicenter collaborative network described previously.^18^ Since the previous report, the network has expanded to include additional institutions, resulting in a larger cohort. We reviewed medical records of patients with SLE who underwent kidney biopsy between January 2003 and August 2023. Patients were eligible if they were aged ≥ 18 years, fulfilled at least 4 of the 1997 American College of Rheumatology criteria^19^ or met the 2019 EULAR/American College of Rheumatology classification criteria,^20^ and were diagnosed with proliferative LN (class III or class IV, with or without class V) based on the International Society of Nephrology and Renal Pathology Society classification.^21^^,^^22^

This study was conducted as a secondary analysis of a multicenter cohort study coordinated by Kobe City Medical Center General Hospital, which obtained central institutional review board approval for data collection and primary analyses (Approval No. zh250702). Fujita Health University participated as one of the collaborating institutions, and the present secondary analysis was additionally reviewed and approved through an expedited review process by the ethics review board of Fujita Health University (Approval No. EN25-172). The study adhered to the principles of the Declaration of Helsinki. Given the retrospective nature of the study and the use of existing clinical data, an opt-out approach for informed consent was implemented in accordance with institutional and national ethical guidelines.

### Data Collection

Baseline demographic and clinical data were collected at the time of kidney biopsy, including age, sex, histological findings, and laboratory parameters such as UPCR (g/g), eGFR (ml/min per 1.73 m^2^), and complement C3 and C4 levels. eGFR was calculated using the equation for the Japanese population.^23^ Laboratory data, including UPCR and eGFR, were collected before treatment initiation and at 3, 6, 12, and 24 months (windows: ±1, ±1, ±1.5, and ±1.5 months, respectively). When multiple measurements were available within a given window, the value closest to the target visit was used. Treatment information included the initial oral glucocorticoid (GC) dose, expressed as prednisolone-equivalent dose (mg/kg/d), and the use of intravenous GC pulse therapy at induction. In addition, the administration of immunosuppressive agents, such as cyclophosphamide or mycophenolate mofetil, as well as hydroxychloroquine was recorded. Presence of chronic lesions was defined as an A/C or C subdivision of class III or IV under the 2003 International Society of Nephrology and Renal Pathology Society classification, as recorded in the original pathology report.

### Definitions of EULAR-Recommended Treatment Targets and Study Outcomes

Treatment response, as defined by the EULAR recommendations, was used as the primary predictor. Patients were classified according to achievement of the EULAR-recommended treatment targets.^10^^,^^11^ Achievement of the 3-month target was defined as a ≥ 25% reduction in UPCR with preserved kidney function (eGFR ≥ 80% of baseline), and achievement of the 6-month target as a ≥ 50% reduction in UPCR. For transition analyses only, achievement of the 12-month target was defined as a UPCR < 700 mg/g. Patients achieving these targets are hereafter termed “3-month target achievers” and “6-month target achievers,” respectively.

The primary outcome was defined as a ≥ 30% decline in eGFR from the higher of the eGFR values measured at baseline (month 0) and at month 3 (hereafter referred to as the “3-month peak eGFR”) to the value at 24 months. Longitudinal eGFR trajectories over 24 months were additionally evaluated using linear mixed-effects models. Because eGFR at presentation may be reduced by potentially reversible inflammatory kidney injury and may improve during induction therapy, the higher value was selected to capture subsequent kidney function loss from the best observed eGFR during the presentation-to-early-induction period.

### Statistical Analysis

All analyses were conducted as complete-case analyses. The 3-month analytic cohort was defined by the availability of 3-month EULAR response status, baseline covariates, and the primary outcome. This cohort was used for the 3-month logistic regression and mixed-effects analyses. The 6-month analytic cohort comprised the subset of the 3-month analytic cohort with available 6-month response assessment. For transition analyses, the Sankey diagram was restricted to patients with available response-status data at 3, 6, and 12 months and an evaluable 24-month eGFR outcome, and the additional transition-based logistic regression analysis was performed in patients with available 3- and 6-month response data.

Baseline characteristics were summarized as mean ± standard deviation or number (percentage). Included and excluded patients were compared using standardized mean differences.

Primary association analyses were performed using multivariable logistic regression for the 3-month and 6-month EULAR response definitions, using response status at each time point as the exposure and a ≥ 30% decline in eGFR at 24 months from the 3-month peak eGFR as the outcome. Overall, 3 sequential models were fitted. Model 1 was adjusted for sex and age; model 2 was additionally adjusted for baseline UPCR and baseline eGFR; and model 3 was further adjusted for the presence of chronic lesions. Adjusted probabilities were estimated from model 3. In component analyses of the 3-month target, the UPCR-reduction and eGFR-preservation criteria were analyzed separately and jointly using the same sequential models. Alternative eGFR-anchor analyses used baseline-, 3-month-, and 6-month-referenced outcomes. The landmark analyses assessed prognostic value beyond kidney status at each landmark. Alternative eGFR-anchor analyses included a baseline-referenced outcome analysis, in which the outcome was defined as a ≥ 30% decline in eGFR from baseline to 24 months, and 3- and 6-month landmark analyses, in which the outcome was defined as a ≥ 30% decline in eGFR from the respective landmark to 24 months. The 3-month landmark models sequentially adjusted for age, sex, baseline UPCR, 3-month eGFR, and chronic lesions and additionally evaluated the UPCR-reduction criterion; the 6-month landmark models sequentially adjusted for age, sex, 6-month eGFR, and 6-month UPCR. Robustness analyses of the primary association models included replacing the presence of chronic lesions, a histologic factor associated with adverse long-term renal outcomes in LN,^24^, ^25^, ^26^, ^27^, ^28^ with intravenous GC pulse therapy during the initial treatment phase or mixed proliferative/membranous histology (class III/IV + V). Additional robustness analyses used stabilized inverse probability weights for complete-case inclusion estimated from the model 3 covariates and treatment era and truncated at the 1st and 99th percentiles, further adjusted model 3 for treatment era, and estimated center-clustered robust standard errors. Supplementary Table S1 summarizes these analyses.

The longitudinal course of kidney function was evaluated using linear mixed-effects models with post-baseline eGFR at 3, 6, 12, and 24 months as the dependent variable. Fixed effects included visit month, treatment response status (3-month or 6-month EULAR response, depending on the model), their interaction, sex, age, baseline UPCR, baseline eGFR, and the presence of chronic lesions; separate models were fitted for the 3-month and 6-month response definitions. Patient-level random intercepts were included to account for within-patient correlation. Marginal predictions were obtained at each post-baseline visit with continuous covariates fixed at their mean values and categorical covariates fixed at their most frequent category. Adjusted change from baseline was derived by subtracting the mean baseline eGFR from the predicted post-baseline eGFR values. For the 6-month response model, the estimate at 3 months was retained as a descriptive preclassification comparison, whereas interpretation of postclassification differences focused on the 6-, 12-, and 24-month visits. As a sensitivity analysis, the mixed-effects analysis from 3 months onward was repeated using a modified 3-month response definition retaining only the UPCR criterion.

Transitions in EULAR response status at 3, 6, and 12 months were visualized using a Sankey diagram together with the occurrence of the primary outcome at 24 months. To further evaluate the prognostic importance of response timing, an additional multivariable logistic regression model classified patients as having achieved the target by 3 months, first achieved it at 6 months, or not achieved it by 6 months, using the same dependent variable and covariates as the fully adjusted primary association model.

All significance tests were 2-sided, with *P* < 0.05 considered statistically significant. All statistical analyses were performed using Python (version 3.12.10; Python Software Foundation, Beaverton, OR) and relevant packages including statsmodels and scikit-learn.

## Results

### Patient Selection and Baseline Clinical Characteristics

Of 449 identified patients, 156 patients were excluded because age was <18 years (*n* = 32) or data required for the 3-month analytic cohort (*n* = 124) were missing. Baseline characteristics were broadly similar between patients included in and excluded from the 3-month analytic cohort (Supplementary Table S2). The remaining 293 patients constituted the 3-month analytic cohort (Figure 1). Among these 293 patients, those with available 6-month response data were analyzed as the 6-month analytic cohort (*n* = 288).

**Figure 1:**
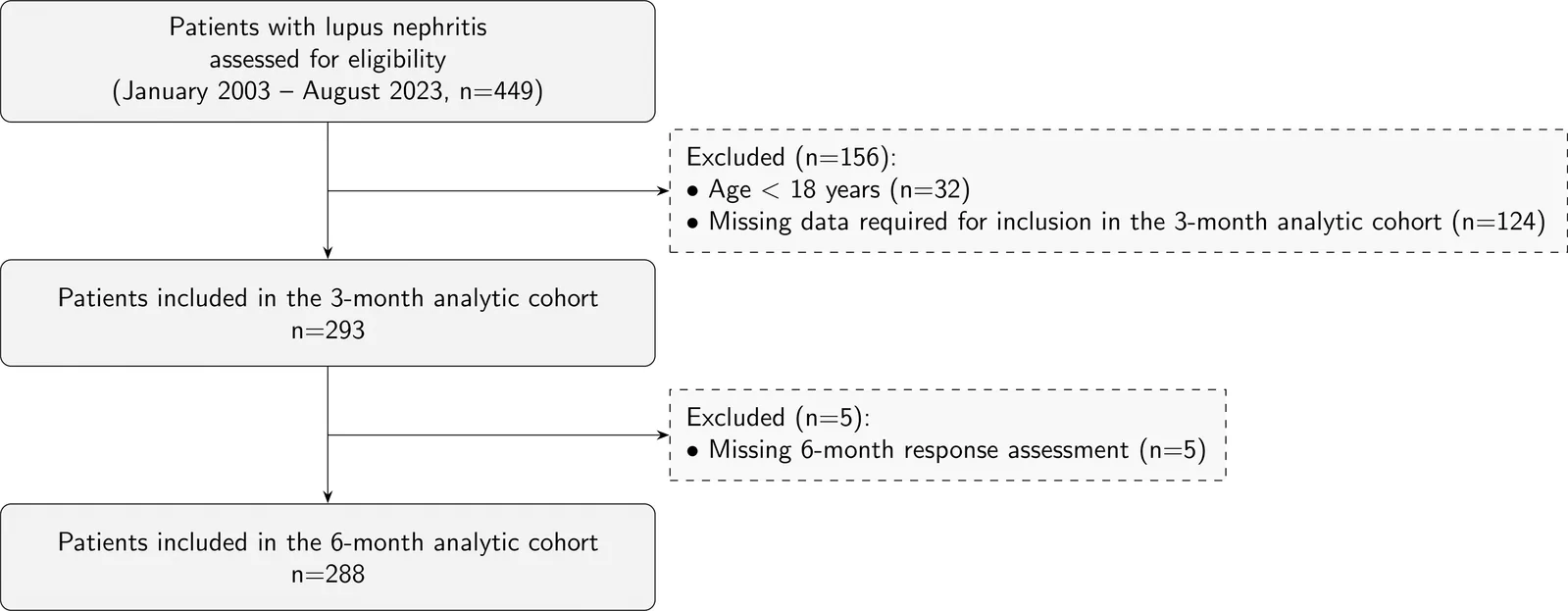
Study flow diagram and definition of the analytic cohorts. Flow diagram of patient selection and analytic cohort definition. Among 449 patients initially identified, 156 were excluded because of age < 18 years (*n* = 32) or missing data required for inclusion in the 3-month analytic cohort (*n* = 124). The remaining 293 patients constituted the 3-month analytic cohort used for the 3-month analyses. Among these, 288 patients with available 6-month response assessment were included in the 6-month analytic cohort.

Baseline characteristics of the 3-month analytic cohort are summarized in Table 1. The mean age was 40.3 ± 15.7 years, and 83.3% of the patients were female. The mean baseline eGFR was 75.4 ± 33.1 ml/min per 1.73 m^2^, and the mean UPCR was 3.3 ± 3.5 g/g. Complement levels were reduced at diagnosis (C3: 43.8 ± 22.9 mg/dl; C4: 7.2 ± 7.0 mg/dl), and the mean SLE disease activity index score was 20.6 ± 7.2, indicating high disease activity. Intravenous GC pulse therapy was administered in 51.9% of patients, and 75.4% received cyclophosphamide or mycophenolate mofetil during initial therapy. Hydroxychloroquine was initiated within 6 months in 38.6% of patients.

**Table 1:** Baseline characteristics of the analytic cohorts according to achievement of the EULAR treatment targets at 3 and 6 months

Variable	All	3-mo target		All	6-mo target	
		Achievers	Nonachievers		Achievers	Nonachievers
*n*	293	213	80	288	246	42
Age (yr)	40.3 ± 15.7	40.1 ± 15.6	40.8 ± 16.0	40.5 ± 15.8	39.8 ± 15.6	44.5 ± 16.4
Female	244 (83.3%)	174 (81.7%)	70 (87.5%)	239 (83.0%)	206 (83.7%)	33 (78.6%)
BMI	21.7 ± 3.5	21.7 ± 3.5	21.6 ± 3.4	21.7 ± 3.5	21.5 ± 3.3	22.8 ± 4.5
SLEDAI	20.6 ± 7.2	20.6 ± 7.1	20.5 ± 7.5	20.6 ± 7.2	20.8 ± 7.0	19.5 ± 8.3
Laboratory data						
eGFR (ml/min per 1.73 m^2^)	75.4 ± 33.1	71.3 ± 30.4	86.3 ± 37.4	75.2 ± 33.2	75.5 ± 32.2	73.7 ± 39.3
UPCR (g/g)	3.3 ± 3.5	3.6 ± 3.5	2.6 ± 3.2	3.3 ± 3.5	3.5 ± 3.6	1.9 ± 2.5
C3 (mg/dl)	43.8 ± 22.9	42.3 ± 23.1	47.8 ± 22.1	44.0 ± 23.0	43.5 ± 23.1	46.7 ± 22.5
C4 (mg/dl)	7.2 ± 7.0	7.1 ± 7.3	7.7 ± 6.3	7.3 ± 7.1	7.2 ± 7.3	7.9 ± 5.6
Histological findings						
Membranous mixed LN	102 (34.8%)	72 (33.8%)	30 (37.5%)	100 (34.7%)	82 (33.3%)	18 (42.9%)
Presence of chronic lesions	142 (48.5%)	98 (46.0%)	44 (55.0%)	140 (48.6%)	114 (46.3%)	26 (61.9%)
Treatment						
Use of HCQ	113 (38.6%)	85 (39.9%)	28 (35.0%)	111 (38.5%)	99 (40.2%)	12 (28.6%)
Intravenous GC pulse therapy	152 (51.9%)	116 (54.5%)	36 (45.0%)	149 (51.7%)	129 (52.4%)	20 (47.6%)
Initial prednisolone-equivalent dose (mg/kg/d)	0.9 ± 0.2	0.9 ± 0.2	0.9 ± 0.2	0.9 ± 0.2	0.9 ± 0.2	0.8 ± 0.2
Use of CY or MMF	221 (75.4%)	164 (77.0%)	57 (71.2%)	216 (75.0%)	183 (74.4%)	33 (78.6%)

BMI, body mass index; CY, cyclophosphamide; eGFR, estimated glomerular filtration rate; EULAR, European Alliance of Associations for Rheumatology; HCQ, hydroxychloroquine; GC, glucocorticoid; LN, lupus nephritis; MMF, mycophenolate mofetil; *n*, number; SD, standard deviation; SLEDAI, Systemic Lupus Erythematosus Disease Activity Index; UPCR, urine protein-to-creatinine ratio; yr, year(s).

Data are presented as mean ± SD or n (%). The 3- and 6-month analyses were performed in the 3-month analytic cohort (*n* = 293) and the 6-month analytic cohort (*n* = 288), respectively. Treatment variables were recorded during the initial treatment phase.

### Kidney Outcomes and Changes in Kidney Function According to Early Treatment Response

Kidney outcomes according to achievement of the EULAR 3- and 6-month treatment targets are shown in Table 2. In the 3-month analysis, a ≥ 30% decline in eGFR from the 3-month peak to 24 months occurred in 24 of 213 patients (11.3%) among 3-month target achievers and in 18 of 80 patients (22.5%) among 3-month target nonachievers. In the 6-month analysis, the corresponding proportions were 31 of 246 patients (12.6%) and 11 of 42 patients (26.2%), respectively. Crude longitudinal UPCR and eGFR values are summarized in Supplementary Table S3, with separate stratification by EULAR target achievement at 3 and 6 months and by occurrence of the primary kidney outcome at 24 months.

**Table 2:** Kidney outcomes and multivariable associations of early EULAR treatment response and components of the 3-month target with a ≥ 30% decline in eGFR at 24 months

Analysis	Response definition	Achievers, *n*	Nonachievers, *n*	Outcome in achievers, *n* (%)	Outcome in nonachievers, *n* (%)	Adjusted OR for target achievement (95% CI)	*P* value
**Primary association analyses**							
	3-mo EULAR target	213	80	24 (11.3)	18 (22.5)	0.40 (0.20–0.82)	0.013
	6-mo EULAR target	246	42	31 (12.6)	11 (26.2)	0.40 (0.17–0.91)	0.030

	**Criterion**	**Patients meeting criterion, *n***	**Patients not meeting criterion, *n***	**Outcome in patients meeting criterion, *n* (%)**	**Outcome in patients not meeting criterion, *n* (%)**	**Adjusted OR for meeting the criterion (95% CI)**	***P* value**
**3-mo component analyses: criteria entered separately**							
	UPCR reduction ≥ 25% at 3 mo	249	44	32 (12.9)	10 (22.7)	0.45 (0.19–1.04)	0.060
	eGFR preservation ≥ 80% of baseline at 3 mo	254	39	33 (13.0)	9 (23.1)	0.45 (0.17–1.17)	0.100
**3-mo component analysis: criteria entered simultaneously**							
	UPCR reduction ≥ 25% at 3 mo	249	44	32 (12.9)	10 (22.7)	0.42 (0.18–0.98)	0.045
	eGFR preservation ≥ 80% of baseline at 3 mo	254	39	33 (13.0)	9 (23.1)	0.42 (0.16–1.09)	0.075

CI, confidence interval; eGFR, estimated glomerular filtration rate; EULAR, European Alliance of Associations for Rheumatology; mo, month; OR, odds ratio; UPCR, urine protein-to-creatinine ratio.

Adjusted ORs were estimated using Model 3, which was adjusted for sex, age, baseline UPCR, baseline eGFR, and the presence of chronic lesions. For the primary association analyses, the 3- and 6-month analyses were performed in the 3-month analytic cohort (*n* = 293) and the 6-month analytic cohort (*n* = 288), respectively. In the component analyses with criteria entered separately, the UPCR-reduction and eGFR-preservation criteria were entered separately into Model 3. In the component analysis with criteria entered simultaneously, both criteria were entered simultaneously into Model 3. Descriptive counts and outcome frequencies are shown for each criterion and are therefore identical in the separate and simultaneous component analyses. Detailed results for Models 1-3 of the primary association analyses are shown in Supplementary Table S4, and detailed results for the component analyses are shown in Supplementary Table S5.

In the primary association analyses, achievement of the 3-month target was associated with lower odds of the outcome across all models, with ORs ranging from 0.39 to 0.42 (Table 2). In model 3, the adjusted probability of the outcome was 11.1% (95% CI, 7.3–15.8) in 3-month target achievers and 23.0% (95% CI, 13.9–33.1) in nonachievers, corresponding to an absolute risk difference of 11.9 (95% CI, 0.9–23.4) percentage points. Likewise, achievement of the 6-month target was consistently associated with lower odds of the outcome, with ORs ranging from 0.38 to 0.45 across models (Table 2). In model 3, the adjusted probability of the outcome was 12.6% (95% CI, 8.6–16.9) in 6-month target achievers and 25.9% (95% CI, 12.7–41.5) in nonachievers, corresponding to an absolute risk difference of 13.3 (95% CI, −0.8 to 29.8) percentage points.

In the component analyses of the 3-month target, the UPCR-reduction and eGFR-preservation criteria of the 3-month target showed similar point estimates (OR, 0.45; 95% CI, 0.19–1.04; *P* = 0.060 and OR, 0.45; 95% CI, 0.17–1.17; *P* = 0.100, respectively; Table 2). When both criteria were entered simultaneously, the corresponding ORs were 0.42 (95% CI, 0.18–0.98; *P* = 0.045) and 0.42 (95% CI, 0.16–1.09; *P* = 0.075), respectively (Table 2). Detailed model results are shown in Supplementary Tables S4 and S5.

Next, we performed alternative eGFR-anchor analyses. With an outcome defined as a ≥ 30% decline in eGFR from baseline to 24 months, the fully adjusted estimates for the 3- and 6-month targets remained below 1.0 but were not statistically significant (OR, 0.43; 95% CI, 0.15–1.26; *P* = 0.126, and OR, 0.48; 95% CI, 0.15–1.54; *P* = 0.215, respectively; Supplementary Table S6, Panel A). In the 3-month landmark analysis, the association of the composite 3-month target was attenuated and no longer statistically significant (OR, 0.75; 95% CI, 0.31–1.76; *P* = 0.504), whereas the UPCR-reduction criterion remained associated with lower odds of subsequent eGFR decline (OR, 0.34; 95% CI, 0.14–0.86; *P* = 0.022; Supplementary Table S6, Panels B and C). In the 6-month landmark analysis, the fully adjusted estimate was close to the null with a wide CI (OR, 1.00; 95% CI, 0.25–4.06; *P* = 0.999; Supplementary Table S6, Panel D).

In robustness analyses of the primary associations, replacing the presence of chronic lesions in model 3 with either intravenous GC pulse therapy during the initial treatment phase or mixed proliferative and membranous histology (class III/IV + V) yielded similar estimates for both the 3- and 6-month targets (Supplementary Table S7). Additional adjustment for treatment-related variables, including initial prednisolone-equivalent dose, cyclophosphamide or mycophenolate mofetil use, hydroxychloroquine use, calcineurin inhibitor use, or belimumab use, also yielded similar estimates. However, these analyses should be interpreted cautiously because treatment information was collected during the initial 6 months and may not strictly represent baseline exposure. The estimates were also similar after inverse probability weighting for complete-case inclusion and additional adjustment for treatment era. Use of center-clustered robust standard errors did not alter the point estimates, although the 95% CI for the 6-month target included the null (*P* = 0.051; Supplementary Table S8).

### eGFR Trajectories According to Treatment Response at 3 and 6 Months

In mixed-effects models, target achievers showed consistently more favorable adjusted eGFR trajectories than nonachievers. In the 3-month analysis, adjusted between-group difference in eGFR significantly favored 3-month target achievers at all evaluated visits, ranging from approximately 10–14 ml/min per 1.73 m^2^ across follow-up (all *P* < 0.001; Table 3). In the 6-month analysis, adjusted eGFR was already higher at 3 months in patients who subsequently achieved the 6-month target, indicating preclassification separation. From 6 months onward, adjusted eGFR remained significantly higher in 6-month target achievers than in nonachievers, with between-group differences of approximately 12–16 ml/min per 1.73 m^2^ at 6, 12, and 24 months (all *P* < 0.001; Table 3). Figure 2 shows the adjusted mean change in eGFR from baseline over time, demonstrating an early separation between achievers and nonachievers that persisted through 24 months in both analyses. Supplementary Figure S1 presents the corresponding model-estimated eGFR trajectories as absolute values.

**Table 3:** Adjusted difference in eGFR between target achievers and non-achievers at each post-baseline visit

Visit	Adjusted difference in eGFR between target achievers and nonachievers (β, mL/min/1.73 m^2^)	95% CI	*P* value
**3-mo treatment response**			
3 mo	14.36	10.12–18.60	< 0.001
6 mo	13.49	9.26–17.72	< 0.001
12 mo	10.81	6.59–15.04	< 0.001
24 mo	10.34	6.12–14.57	< 0.001
**6-mo treatment response**			
3 mo	10.47	6.27–14.66	< 0.001
6 mo	15.80	11.61–19.99	< 0.001
12 mo	12.23	8.04–16.42	< 0.001
24 mo	12.17	7.98–16.36	< 0.001

CI, confidence interval; eGFR, estimated glomerular filtration rate; mo, month.

Values are presented as adjusted between-group differences in eGFR (β) from linear mixed-effects models. Separate models were fitted for the 3-month and 6-month response definitions. Models included visit month, treatment response status, their interaction, sex, age, baseline UPCR, baseline eGFR, and the presence of chronic lesions, with patient-level random intercepts. Positive β values indicate higher adjusted eGFR in target achievers than in non-achievers. In the 6-month treatment response analysis, the 3-month estimate represents a descriptive between-group difference before classification of 6-month response status; interpretation of the 6-month response analysis therefore focuses on the 6-, 12-, and 24-month estimates.

**Figure 2:**
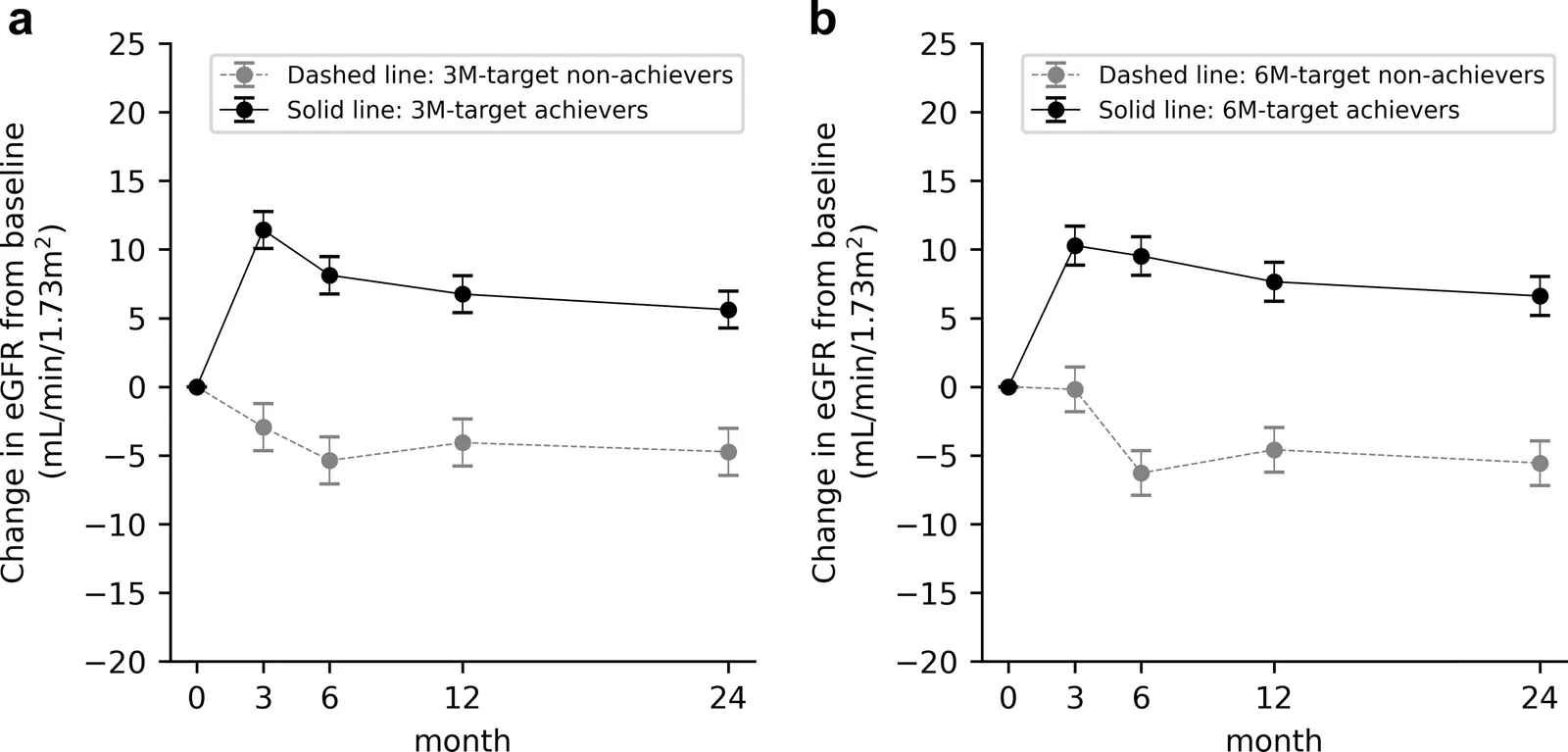
Adjusted change in eGFR from baseline according to achievement of the EULAR treatment targets at 3 and 6 months. Adjusted change in eGFR from baseline over time according to achievement of the EULAR treatment targets at 3 months (a) and 6 months (b). Values were estimated from linear mixed-effects models adjusted for sex, age, baseline UPCR, baseline eGFR, and the presence of chronic lesions. Separate models were fitted for the 3-month and 6-month response definitions. Error bars indicate standard errors. In panel b, the 3-month value represents a descriptive preclassification estimate because 6-month response status was defined at 6 months. eGFR, estimated glomerular filtration rate; EULAR, European Alliance of Associations for Rheumatology; 3M, 3 months; 6M, 6 months; UPCR, urine protein-to-creatinine ratio.

Model-estimated eGFR trajectories were materially similar in a sensitivity analysis in which the presence of chronic lesions was replaced by intravenous GC pulse therapy during the initial treatment phase (Supplementary Figure S2). In a sensitivity analysis using the UPCR-reduction criterion alone, adjusted between-group differences in eGFR change from 3 months onward were attenuated; however, achievers still showed significantly more favorable changes in eGFR from 3 months onward (Supplementary Table S9).

### Patient Transitions in EULAR Response Status Over Time

Figure 3 summarizes longitudinal transitions in EULAR response status at 3, 6, and 12 months among the 288 patients with available response data at all 3 time points and an evaluable 24-month eGFR outcome. At 3 months, 210 patients (72.9%) had achieved the 3-month target and 78 (27.1%) had not. Among the 78 patients who did not achieve the 3-month target, 51 subsequently met the 6-month target, whereas 27 did not meet the 6-month target. Of these 27 patients, 17 met the 12-month target and 10 remained nonachievers throughout the first 12 months. The proportion of patients with a ≥ 30% decline in eGFR at 24 months showed a graded increase according to the timing of first target achievement: 11.4% among those who achieved the 3-month target at 3 months, 19.6% among those who first achieved the 6-month target, 23.5% among those who first achieved the 12-month target, and 40.0% among those who never achieved any target. A more detailed Sankey diagram showing all transition pathways is provided in Supplementary Figure S3.

**Figure 3:**
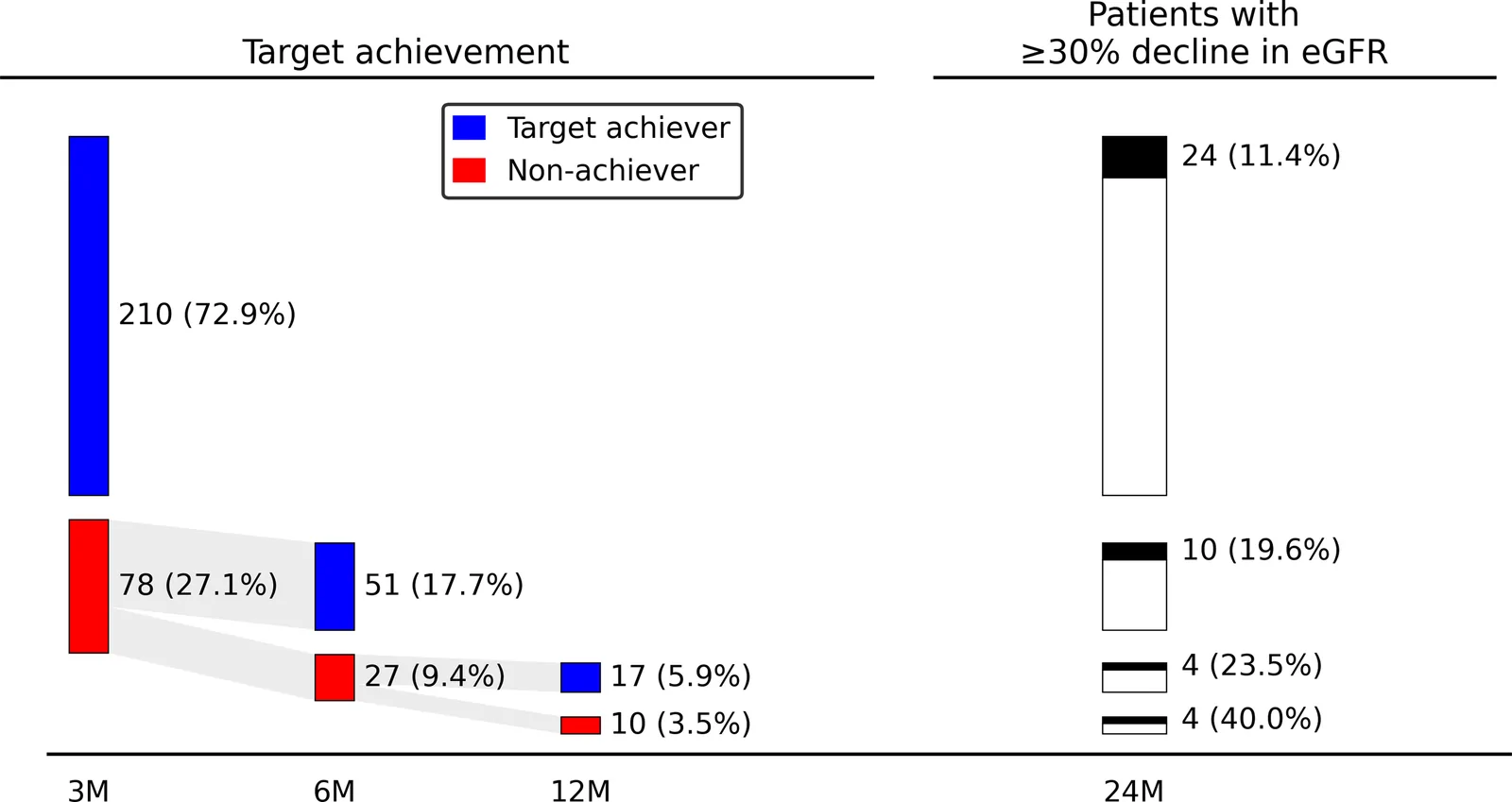
Longitudinal transitions in EULAR treatment response status and subsequent risk of a ≥ 30% decline in eGFR at 24 months. Sankey diagram shows transitions in EULAR treatment response status at 3, 6, and 12 months and the proportion of patients with a ≥ 30% decline in eGFR at 24 months. The analysis included patients with available response-status data at all 3 time points and an evaluable 24-month eGFR outcome. The widths of the flows are proportional to the number of patients in each transition pathway. Percentages shown at the final nodes indicate the proportion of patients with a ≥ 30% decline in eGFR at 24 months according to the timing of first achievement of the EULAR treatment target. eGFR, estimated glomerular filtration rate; EULAR, European Alliance of Associations for Rheumatology; 3M, 3 months; 6M, 6 months; 12M, 12 months; 24M, 24 months.

To further assess the prognostic importance of the timing of response achievement, we performed an additional multivariable logistic regression analysis classifying patients as having achieved the target by 3 months, first achieved it at 6 months, or not achieved it by 6 months (Figure 4). Compared with patients who had not achieved the target by 6 months, those who had already achieved it by 3 months had substantially lower adjusted odds of a ≥ 30% decline in eGFR at 24 months (OR, 0.26; 95% CI, 0.10–0.70). Patients who first achieved the target at 6 months also showed a lower point estimate for risk, although the association did not reach statistical significance (OR, 0.52; 95% CI, 0.17–1.59). Together, these findings suggest that achievement of the EULAR treatment target by 3 months was associated with the most favorable renal prognosis.

**Figure 4:**
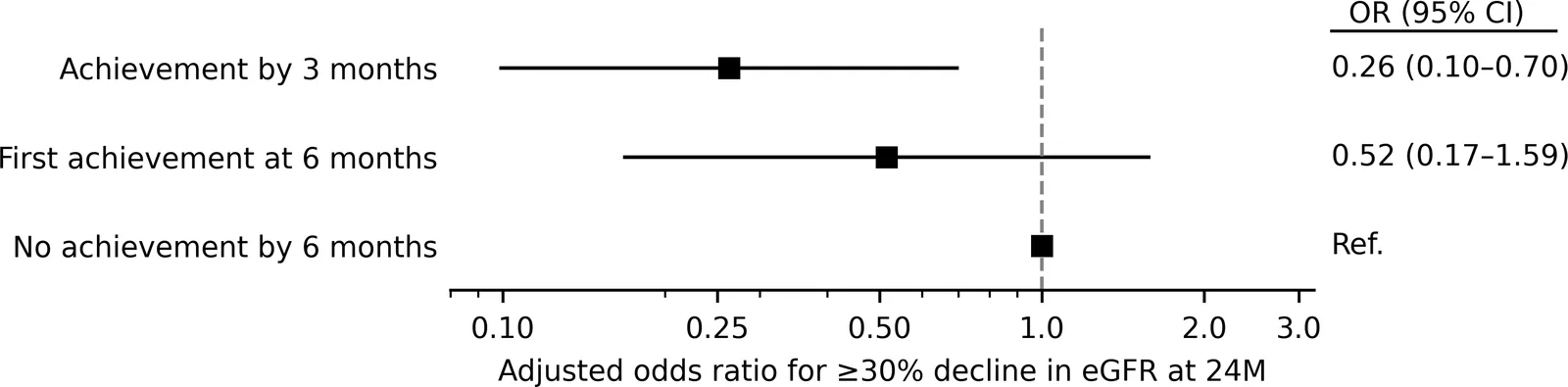
Adjusted odds of a ≥ 30% decline in eGFR at 24 months according to the timing of achievement of the EULAR treatment target. Adjusted odds ratios for a ≥ 30% decline in eGFR at 24 months according to the timing of achievement of the EULAR treatment target. Patients were classified as having achieved the target by 3 months, first achieved the target at 6 months, or not achieved the target by 6 months. Odds ratios were estimated using a multivariable logistic regression model adjusted for sex, age, baseline UPCR, baseline eGFR, and the presence of chronic lesions, with patients who had not achieved the target by 6 months as the reference group. Error bars indicate 95% CIs. CI, confidence interval; eGFR, estimated glomerular filtration rate; EULAR, European Alliance of Associations for Rheumatology; OR, odds ratio; UPCR, urine protein-to-creatinine ratio.

## Discussion

In this study, we evaluated whether early treatment responses defined according to the EULAR recommendations were associated with subsequent decline in kidney function in patients with LN. In multivariable logistic regression analyses, achievement of the 3- or 6-month EULAR response was associated with substantially lower adjusted odds of a ≥ 30% decline in eGFR at 24 months, with absolute risk differences of approximately 12–13 percentage points between achievers and nonachievers. Consistent with these findings, linear mixed-effects models showed that both 3- and 6-month responders had higher adjusted eGFR trajectories over follow-up than nonresponders. These associations persisted after adjustment for baseline clinical and histological factors, supporting early response, particularly at 3 months, as a marker for short-term risk stratification.

Building on prior work, which has demonstrated that failure to achieve the 6-month EULAR response predicts long-term CKD progression and end-stage kidney disease,^12^^,^^13^^,^^15^ our findings extend this evidence by showing that EULAR-based treatment targets already differentiated risk within a much shorter timeframe. Moreover, the early between-group difference in eGFR persisted through 6, 12, and 24 months, suggesting that early target achievement may identify patients with more favorable subsequent renal trajectories.

Mechanistically, persistent proteinuria may both reflect residual disease activity and contribute to progressive kidney dysfunction. Tubular uptake of filtered proteins activates inflammatory and profibrotic pathways. In parallel, ongoing glomerular injury with podocyte loss may promote maladaptive hyperfiltration. Together, these processes lead to tubulointerstitial fibrosis, irreversible nephron loss, and ultimately GFR decline.^29^ Accordingly, early proteinuria reduction may indicate more rapid attenuation of these injurious processes, consistent with its association with better kidney outcomes at 24 months.

The Sankey diagram illustrated heterogeneous response trajectories, broadly consistent with recent real-world data on EULAR/European Renal Association–European Dialysis and Transplant Association target attainment in LN.^30^ Most patients who achieved the 3-month response remained responders at 6 and 12 months and had a correspondingly low proportion of subsequent ≥ 30% eGFR decline at 24 months, whereas many 3-month nonachievers subsequently achieved a target but remained at higher risk than early achievers. Consistently, the transition-based analysis suggested that achievement by 3 months was associated with the most favorable risk profile. These findings support the 3-month assessment for early risk stratification, with reassessment at 6 months helping to further refine risk classification.

From a clinical perspective, failure to achieve the 3-month target may identify a higher-risk subgroup in whom closer monitoring and evaluation of treatment adequacy may be warranted; however, this study did not assess whether treatment modification based on early target status improves outcomes.

Because the 3-month EULAR target includes an eGFR-preservation component and the primary outcome was referenced to the higher of baseline or 3-month eGFR, this partial overlap may introduce mathematical coupling and built-in prognostic separation. Component analyses yielded similar point estimates for the UPCR-reduction and eGFR-preservation criteria and did not support attributing the association solely to either component. However, in the 3-month landmark analysis, the composite association was attenuated after adjustment for 3-month eGFR, whereas the UPCR-reduction criterion remained associated with subsequent eGFR decline. Thus, the observed association may partly reflect kidney function already evident at 3 months, although early proteinuria reduction retained prognostic relevance beyond 3-month eGFR. For the 6-month target, preclassification eGFR separation and the imprecise landmark estimate leave its incremental prognostic value beyond kidney status at 6 months uncertain.

We selected a ≥ 30% eGFR decline as a clinically meaningful intermediate endpoint suitable for the 24-month follow-up period.^31^^,^^32^ However, using the higher of baseline or 3-month eGFR as the reference may be susceptible to regression to the mean. The associations were attenuated and no longer statistically significant when baseline eGFR alone was used as the reference, although the estimates were imprecise because only 18 events occurred.

This study has several limitations. First, due to its retrospective observational design, causality cannot be established, and unmeasured confounding factors, including treatment modifications during the early treatment phase, may have influenced both treatment response and renal outcomes. Second, although treatment-era adjustment did not materially alter the estimates, residual confounding from evolving treatment practices and between-center differences cannot be excluded; center-clustered robust standard errors account for within-center correlation but not systematic center-level differences. Hydroxychloroquine use was low (38.6%), partly reflecting limited availability in Japan during earlier years, which may limit generalizability. Third, although inverse probability weighting yielded results similar to the complete-case analyses, residual selection bias from missing follow-up data or unmeasured determinants of missingness remains possible. In addition, treatment response at each time point was defined using laboratory values obtained within prespecified windows, and variability in visit schedules or testing intervals may have led to misclassification. Fourth, detailed histological measures, including activity and chronicity indices and specific histological lesions, were not consistently available; therefore, residual confounding by histological severity cannot be excluded. Finally, because this study evaluated a 24-month outcome, the longer-term prognostic implications of early response could not be assessed.

This study has important strengths. It was conducted as a multicenter collaboration and included a substantial number of biopsy-proven LN cases, providing a well-characterized dataset for analysis. Treatment response was defined strictly according to EULAR recommendations, enabling assessment of guideline-based targets in real-world practice. Importantly, the study systematically examined early-phase response at 3 months—a time point that has received limited attention in previous research—and showed that this early assessment provides clinically relevant prognostic information.

In conclusion, early treatment response defined according to the EULAR recommendations, particularly at 3 months, was associated with more favorable kidney outcomes through 24 months. Assessment at 3 months may provide early prognostic information for risk stratification.

## Disclosure

RU reports lecture honoraria from Boehringer Ingelheim. HI reports lecture honoraria from Chugai Pharmaceutical, Otsuka Pharmaceutical, and AstraZeneca. K. Hiromura reports research support from Chugai Pharmaceutical and Asahi Kasei Pharma; consulting fees from Otsuka Pharmaceutical, GlaxoSmithKline, and Chugai Pharmaceutical; and lecture honoraria from AstraZeneca, Asahi Kasei Pharma, GlaxoSmithKline, Novartis Pharma, Otsuka Pharmaceutical, and Astellas Pharma. N. Tsuboi reports a consigned research fund from Zenyaku Kogyo; advisory fees from Otsuka Pharmaceutical, Novartis Pharma, and Chugai Pharmaceutical; and lecture fees from Astellas Pharma, AstraZeneca, Chugai Pharmaceutical, GlaxoSmithKline, Novartis Pharma, Otsuka Pharmaceutical, and Sanofi. Y. Kawahito reports grants or contracts from Asahi Kasei Pharma/Asahi Kasei Therapeutics and Chugai Pharmaceutical, and lecture honoraria from Asahi Kasei Pharma, Novartis Pharma, Otsuka Pharmaceutical, Bristol Myers Squibb, Chugai Pharmaceutical, GlaxoSmithKline, and AstraZeneca. TS reports lecture honoraria from Chugai Pharmaceutical, Novartis Pharma, Otsuka Pharmaceutical, Bristol Myers Squibb, Asahi Kasei Therapeutics, GlaxoSmithKline, and AstraZeneca. KK reports speaker fees from Otsuka Pharmaceutical. M. Kono reports grants from Takeda, GlaxoSmithKline, Mitsubishi Tanabe Pharma, Sanofi, Taisho Pharmaceutical, Nippon Shinyaku, Taiju Life Social Welfare Foundation, Kowa, Terumo, Kyocera, Chugai Pharmaceutical, Lotte, Mochida, Hitachi, Yamazaki Baking, and Astellas, and speaker fees from AbbVie, Asahi Kasei, Astellas, AstraZeneca, Ayumi, BioCryst Japan, Bristol Myers Squibb, Chugai Pharmaceutical, Daiichi Sankyo, Eisai, Eli Lilly, Gilead Sciences, GlaxoSmithKline, Janssen, Kowa, Kissei, Kyowa Kirin, Lotte, Nippon Boehringer Ingelheim, Nippon Shinyaku, Mitsubishi Tanabe Pharma, Mochida, Pfizer, Sandoz, Taisho, Takeda, and UCB Japan. AO reports grants or departmental support from Pfizer, Bristol Myers Squibb, Asahi Kasei Pharma, Taisho Pharmaceutical, Nagahama City, and Toyooka City, and speaker bureau fees from Asahi Kasei Pharma, Chugai Pharmaceutical, Eli Lilly Japan, Ono Pharmaceutical, UCB Japan, Mitsubishi Tanabe Pharma, Taisho Pharmaceutical, Eisai, AbbVie, and Daiichi Sankyo. Kentaro Minowa reports honoraria from Asahi Kasei Pharma, GlaxoSmithKline, Otsuka Pharmaceutical, and AstraZeneca. N. Tamura reports grants or contracts from Asahi Kasei Pharma, Nippon Boehringer Ingelheim, Bristol Myers Squibb, Ayumi Pharmaceutical, Chugai Pharmaceutical, and Taisho Pharmaceutical, and lecture honoraria from AstraZeneca, Eli Lilly Japan, Kissei Pharmaceutical, UCB Japan, AbbVie, Otsuka Pharmaceutical, and GlaxoSmithKline. S. Kaname reports grants or contracts from Teijin Pharma, Nippon Boehringer Ingelheim, Asahi Kasei Pharma, Mochida Pharmaceutical, Otsuka Pharmaceutical, and Taisho Pharmaceutical; consulting fees from Sato Pharmaceutical, Japan Blood Products Organization, Kissei Pharmaceutical, and Alexion Pharmaceutical; and lecture honoraria from Teijin Pharma, Kissei Pharmaceutical, AstraZeneca, Teijin Health Care, Chugai Pharmaceutical, Mitsubishi Tanabe Pharma, Kyowa Kirin, Novartis Pharma, Daiichi Sankyo, and Alexion Pharmaceutical. T. Kawakami reports lecture honoraria from Astellas Pharma, Otsuka Pharmaceutical, Novartis Pharma, and AstraZeneca. YT reports a research grant from Asahi Kasei and speaking fees and/or honoraria from AstraZeneca, Chugai Pharmaceutical, Boehringer Ingelheim, Novartis, Otsuka Pharmaceutical, AbbVie, Eli Lilly, UCB, GlaxoSmithKline, Eisai, Sandoz, Bristol Myers Squibb, Gilead, Sanofi, Taisho, Biogen, and Mitsubishi Tanabe Pharma. S. Kubo reports grants from Ono Pharmaceutical Foundation for Oncology, Immunology, and Neurology, the JCR Grant for Promoting Research for FRONTIER, Daiichi Sankyo, AbbVie, Boehringer Ingelheim, and Astellas, and honoraria from Eli Lilly, GlaxoSmithKline, AbbVie, UCB, Pfizer, and Boehringer Ingelheim. Shuji Sumitomo reports speaker honoraria from Asahi Kasei Pharma, Astellas Pharma, AstraZeneca, Bristol Myers Squibb, Chugai Pharmaceutical, GlaxoSmithKline, and Otsuka Pharmaceutical. Y. Kaneko reports grants from Chugai Pharmaceutical and Asahi Kasei Pharma/Asahi Kasei Therapeutics; consulting fees from Chugai Pharmaceutical, Asahi Kasei Pharma/Asahi Kasei Therapeutics, Astellas Pharma, Novartis Pharma, GlaxoSmithKline, Otsuka Pharmaceutical, and AstraZeneca; and honoraria from Chugai Pharmaceutical, Asahi Kasei Pharma/Asahi Kasei Therapeutics, Sanofi, Astellas Pharma, Novartis Pharma, GlaxoSmithKline, Otsuka Pharmaceutical, and AstraZeneca. T. Koga reports lecture honoraria from GlaxoSmithKline, Novartis, and AstraZeneca. H. Sato reports lecture honoraria from Otsuka Pharmaceutical and Novartis Pharma. Tomonori Ishii reports lecture honoraria from Asahi Kasei, GlaxoSmithKline, Pfizer, Janssen Pharmaceutical, AstraZeneca, and Eli Lilly. S. Maruyama reports collaborative research funding from Zenyaku Kogyo, lecture honoraria from AstraZeneca and Otsuka Pharmaceutical, and scholarship donations from Otsuka Pharmaceutical. HD reports grants from AbbVie, Asahi Kasei Pharma, and Chugai Pharmaceutical; consulting fees from AstraZeneca, UCB Pharmaceutical, and Chugai Pharmaceutical; and honoraria from AbbVie, Ayumi Pharmaceutical, Chugai Pharmaceutical, AstraZeneca, Eisai Pharmaceutical, and UCB Pharmaceutical. MA reports honoraria from Asahi Kasei Pharma, Otsuka Pharmaceutical, GlaxoSmithKline, and AstraZeneca. HN reports research funding from Chugai Pharmaceutical and Asahi Kasei Pharma/Asahi Kasei Therapeutics; consulting fees from Asahi Kasei Pharma/Asahi Kasei Therapeutics; and honoraria from Chugai Pharmaceutical, Asahi Kasei Pharma/Asahi Kasei Therapeutics, Sanofi, Shionogi, Astellas Pharma, Novartis Pharma, GlaxoSmithKline, Otsuka Pharmaceutical, AstraZeneca, and Bristol Myers Squibb. SY reports grants or contracts from Shionogi; consulting fees from Chugai Pharmaceutical and Novartis Pharma; and honoraria from Asahi Kasei Therapeutics, Otsuka Pharmaceutical, Chugai Pharmaceutical, and AstraZeneca. TH reports grants from Japan Research Foundation for Clinical Pharmacology, NEC Corporation, and Terumo Foundation for Life Sciences and Arts, and honoraria from Asahi Kasei Pharma/Asahi Kasei Therapeutics, AstraZeneca, Chugai Pharmaceutical, Mitsubishi Tanabe Pharma, Taisho Pharmaceutical, AbbVie, Novartis Pharma, Otsuka Pharmaceutical, Eli Lilly, Eisai, Daiichi Sankyo, and Teijin Home Healthcare. Y. Kondo reports honoraria from Asahi Kasei Pharma/Asahi Kasei Therapeutics, Novartis Pharma, Otsuka Pharmaceutical, Astellas Pharma, GlaxoSmithKline, and AstraZeneca, and advisory board participation for Asahi Kasei Pharma/Asahi Kasei Therapeutics and Novartis Pharma. TG reports consulting fees from Asahi Kasei Pharma, Otsuka Pharmaceutical, and Sanofi. M. Kuwana reports consulting fees from AstraZeneca, Chugai Pharmaceutical, GlaxoSmithKline, and Novartis, and honoraria from Bristol Myers Squibb and Chugai Pharmaceutical. TA reports research or scholarship grants from GlaxoSmithKline, Bristol Myers Squibb, Zenyaku Kogyo, Amgen, Kissei Pharmaceutical, AbbVie, Mitsubishi Tanabe Pharma, Eisai, Nippon Boehringer Ingelheim, Otsuka Pharmaceutical, and Chugai Pharmaceutical; consulting fees from AstraZeneca, Idorsia Pharmaceuticals, Otsuka Pharmaceutical, Gilead Sciences, GlaxoSmithKline, Sanofi, Eli Lilly Japan, Nippon Boehringer Ingelheim, Janssen Pharmaceutical, UCB Japan, Kissei Pharmaceutical, and Novartis Pharma; honoraria from Bristol Myers Squibb, Eli Lilly Japan, Nippon Boehringer Ingelheim, Eisai, AbbVie, Pfizer, and Gilead Sciences; and leadership roles in the Japan College of Rheumatology, Japanese Society for Thrombosis and Hemostasis, Japanese Society for Clinical Rheumatology, Japanese Society of Internal Medicine, Japanese Society of Clinical Immunology, and Japanese Society of Molecular Medicine. KO reports grants or contracts from Chugai Pharmaceutical, honoraria from Asahi Kasei Pharma, Chugai Pharmaceutical, AstraZeneca, and Otsuka Pharmaceutical, and membership in the SLE Guideline Development Subcommittee. HH reports lecture honoraria from Chugai Pharmaceutical, Astellas Pharma, Asahi Kasei Pharma, AstraZeneca, Novartis Pharma, GlaxoSmithKline, and Otsuka Pharmaceutical. The other authors declare no conflicts of interest.

## Supplementary Material

Supplementary File (PDF) **Figure S1.** Adjusted absolute eGFR trajectories according to achievement of the EULAR treatment targets at 3 and 6 months. **Figure S2.** Sensitivity analysis of adjusted change in eGFR from baseline using intravenous glucocorticoid pulse therapy in place of chronic lesions. **Figure S3.** Detailed Sankey diagram of transitions in EULAR treatment response status and subsequent kidney outcome. **Table S1.** Overview of the logistic regression analyses. **Table S2.** Baseline characteristics and response/outcome availability of patients included in and excluded from the 3-month analytic cohort. **Table S3.** Crude longitudinal UPCR and eGFR values stratified by EULAR target achievement and occurrence of the primary kidney outcome at 24 months. **Table S4.** Full multivariable logistic regression models for a ≥30% decline in eGFR at 24 months according to achievement of the EULAR treatment targets at 3 and 6 months. **Table S5.** Full multivariable logistic regression models for the individual criteria of the 3-month EULAR target. **Table S6.** Alternative eGFR-anchor analyses using baseline-referenced and landmark outcomes. **Table S7.** Robustness analyses of multivariable logistic regression models replacing chronic lesions with either intravenous glucocorticoid pulse therapy during initial treatment or mixed proliferative/membranous histology (class III/IV + V). **Table S8.** Robustness analyses using inverse probability weighting, treatment-era adjustment, and center-clustered robust standard errors. **Table S9.** Sensitivity analysis of between-group differences in eGFR change from 3 months onward using a modified 3-month target definition based on the UPCR criterion alone. **STROBE checklist**.

## Footnotes

Supplementary File (PDF)
**Figure S1.** Adjusted absolute eGFR trajectories according to achievement of the EULAR treatment targets at 3 and 6 months. **Figure S2.** Sensitivity analysis of adjusted change in eGFR from baseline using intravenous glucocorticoid pulse therapy in place of chronic lesions. **Figure S3.** Detailed Sankey diagram of transitions in EULAR treatment response status and subsequent kidney outcome. **Table S1.** Overview of the logistic regression analyses. **Table S2.** Baseline characteristics and response/outcome availability of patients included in and excluded from the 3-month analytic cohort. **Table S3.** Crude longitudinal UPCR and eGFR values stratified by EULAR target achievement and occurrence of the primary kidney outcome at 24 months. **Table S4.** Full multivariable logistic regression models for a ≥30% decline in eGFR at 24 months according to achievement of the EULAR treatment targets at 3 and 6 months. **Table S5.** Full multivariable logistic regression models for the individual criteria of the 3-month EULAR target. **Table S6.** Alternative eGFR-anchor analyses using baseline-referenced and landmark outcomes. **Table S7.** Robustness analyses of multivariable logistic regression models replacing chronic lesions with either intravenous glucocorticoid pulse therapy during initial treatment or mixed proliferative/membranous histology (class III/IV + V). **Table S8.** Robustness analyses using inverse probability weighting, treatment-era adjustment, and center-clustered robust standard errors. **Table S9.** Sensitivity analysis of between-group differences in eGFR change from 3 months onward using a modified 3-month target definition based on the UPCR criterion alone. **STROBE checklist**.
