# Supplementary material for: Early EULAR Target Achievement and Kidney Outcomes in Lupus Nephritis
Source: Kidney Int Rep. 2026 Sep 17;11(12):107079. doi: 10.1016/j.ekir.2026.107079 (PMC13738129; doi:10.1016/j.ekir.2026.107079)
Supplement: Supplementary file 1 [file mmc1.pdf]

# Supplemental Material

**Table of Contents**

Supplementary Figure S1: Adjusted absolute eGFR trajectories according to achievement of the EULAR treatment targets at 3 and 6 months ........ p2

Supplementary Figure S2: Sensitivity analysis of adjusted change in eGFR from baseline using intravenous glucocorticoid pulse therapy in place of chronic lesions ........ p3

Supplementary Figure S3: Detailed Sankey diagram of transitions in EULAR treatment response status and subsequent kidney outcome ........ p4

Supplementary Table S1. Overview of the logistic regression analyses ........ p5

Supplementary Table S2. Baseline characteristics and response/outcome availability of patients included in and excluded from the 3-month analytic cohort ........ p7

Supplementary Table S3. Crude longitudinal UPCR and eGFR values stratified by EULAR target achievement and occurrence of the primary kidney outcome at 24 months ........ p9

Supplementary Table S4. Full multivariable logistic regression models for a ≥30% decline in eGFR at 24 months according to achievement of the EULAR treatment targets at 3 and 6 months ........ p11

Supplementary Table S5. Full multivariable logistic regression models for the individual criteria of the 3-month EULAR target ........ p12

Supplementary Table S6. Alternative eGFR-anchor analyses using baseline-referenced and landmark outcomes ........ p14

Supplementary Table S7. Robustness analyses of multivariable logistic regression models replacing chronic lesions with either intravenous glucocorticoid pulse therapy during initial treatment or mixed proliferative/membranous histology (class III/IV + V) ........ p17

Supplementary Table S8. Robustness analyses using inverse probability weighting, treatment-era adjustment, and center-clustered robust standard errors ........ p19

Supplementary Table S9. Sensitivity analysis of between-group differences in eGFR change from 3 months onward using a modified 3-month target definition based on the UPCR criterion alone ........ p21

STROBE checklist ........ p22

**Supplementary Figure S1: Adjusted absolute eGFR trajectories according to achievement of the EULAR treatment targets at 3 and 6 months**

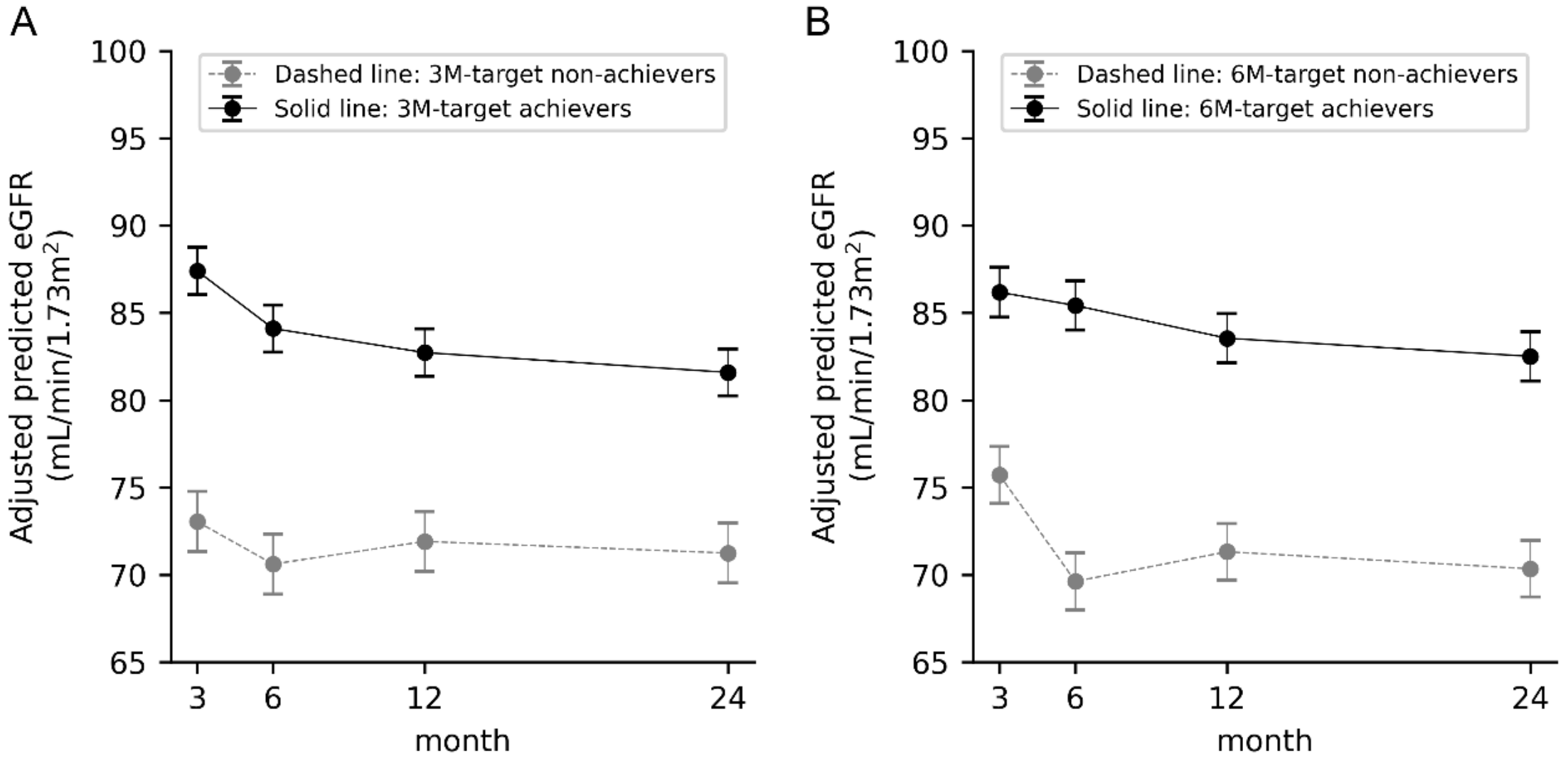


*Legend.* Adjusted absolute eGFR trajectories over time according to achievement of the EULAR treatment targets at 3 months (A) and 6 months (B). Values were estimated from linear mixed-effects models adjusted for sex, age, baseline UPCR, baseline eGFR, and the presence of chronic lesions. Separate models were fitted for the 3-month and 6-month response definitions. Error bars indicate standard errors. In panel B, the 3-month value represents a descriptive pre-classification estimate because 6-month response status was defined at 6 months. eGFR, estimated glomerular filtration rate; UPCR, urine protein-to-creatinine ratio; 3M, 3 month; 6M, 6 month.

**Supplementary Figure S2: Sensitivity analysis of adjusted change in eGFR from baseline using intravenous glucocorticoid pulse therapy in place of chronic lesions**

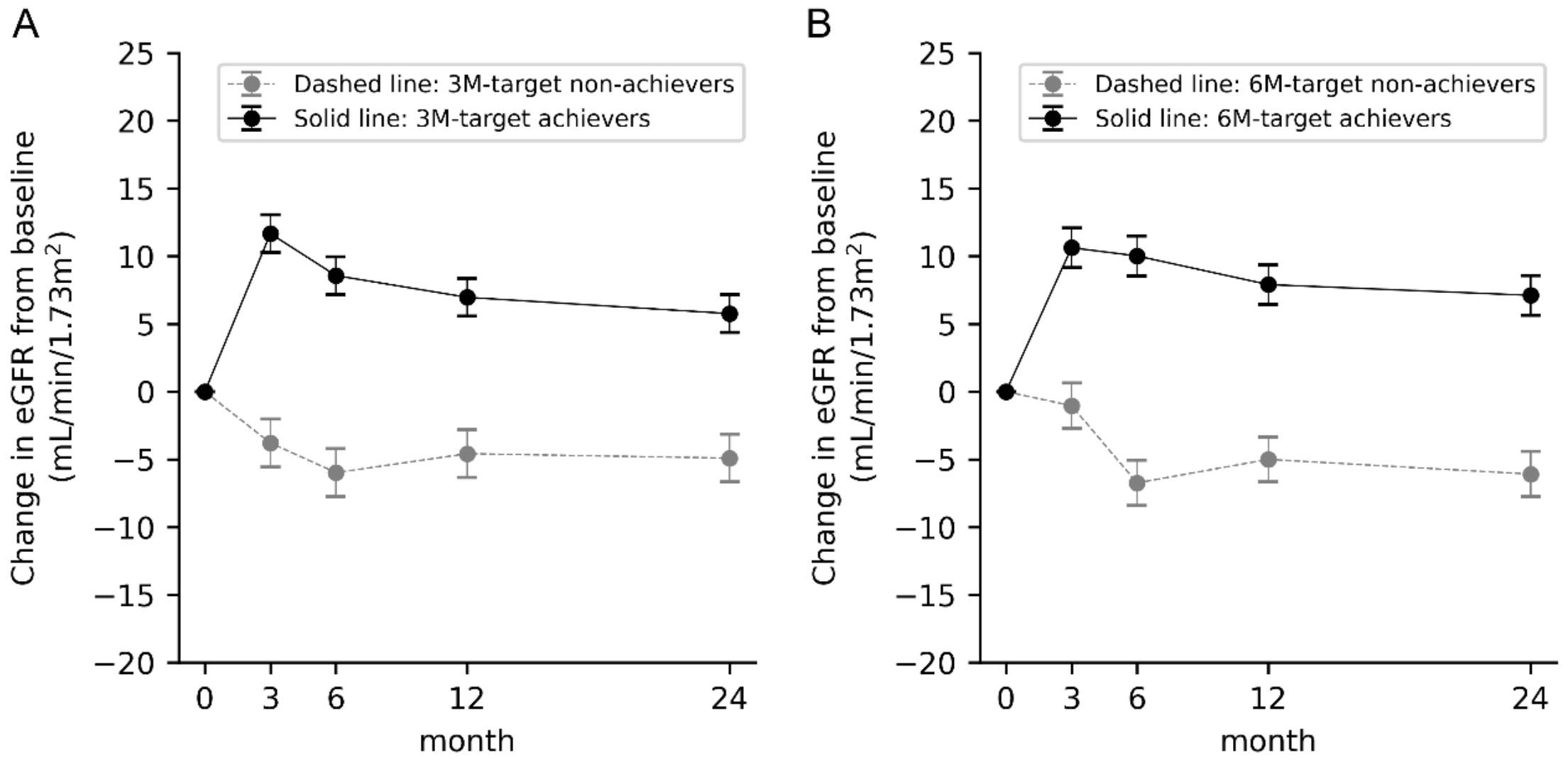


*Legend.* Adjusted change in eGFR from baseline over time according to achievement of the EULAR treatment targets at 3 months (A) and 6 months (B) in a sensitivity analysis in which intravenous glucocorticoid pulse therapy during the initial treatment phase was included in place of the presence of chronic lesions. Values were estimated from linear mixed-effects models adjusted for sex, age, baseline UPCR, baseline eGFR, and intravenous glucocorticoid pulse therapy. Separate models were fitted for the 3-month and 6-month response definitions. Error bars indicate standard errors. In panel B, the 3-month value represents a descriptive pre-classification estimate because 6-month response status was defined at 6 months. eGFR, estimated glomerular filtration rate; UPCR, urine protein-to-creatinine ratio; 3M, 3 month; 6M, 6 month.

**Supplementary Figure S3: Detailed Sankey diagram of transitions in EULAR treatment response status and subsequent kidney outcome**

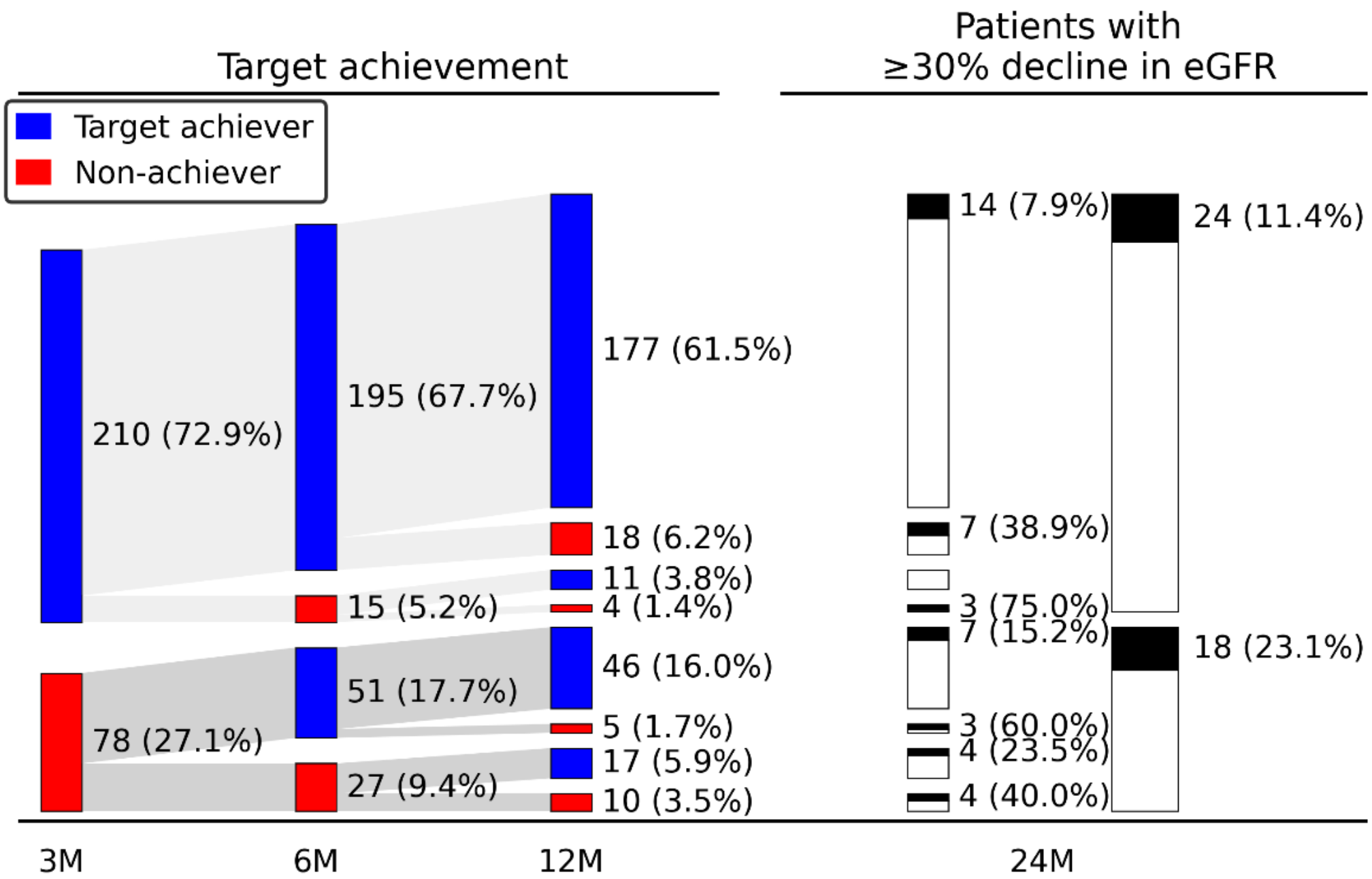


*Legend.* Detailed Sankey diagram showing transitions in EULAR treatment response status at 3, 6, and 12 months and the occurrence of a ≥30% decline in eGFR at 24 months. The analysis included patients with available response-status data at all three time points and an evaluable 24-month eGFR outcome. Flow widths are proportional to the number of patients in each transition pathway. eGFR, estimated glomerular filtration rate.

**Supplementary Table S1. Overview of the logistic regression analyses**

| Analysis family | Analysis | Question addressed | Exposure | eGFR outcome anchor | Key model feature | Cohort | Reported in |
|---|---|---|---|---|---|---|---|
| Primary association | 3-month target | Is 3-month target achievement associated with the primary kidney outcome? | Original 3-month target | Higher of baseline or 3-month eGFR to 24 months | Model 1–3 | 3-month analytic cohort | Table 2; Table S4 |
| Primary association | 6-month target | Is 6-month target achievement associated with the primary kidney outcome? | Original 6-month target | Higher of baseline or 3-month eGFR to 24 months | Model 1–3 | 6-month analytic cohort | Table 2; Table S4 |
| Component | 3-month target components | How are the UPCR-reduction and eGFR-preservation criteria associated with the primary outcome when examined separately and jointly? | UPCR and eGFR criteria separately and jointly | Primary outcome | Model 1–3 | 3-month analytic cohort | Table 2; Table S5 |
| Alternative eGFR anchor | Baseline-referenced analysis | Are findings dependent on use of the 3-month peak eGFR? | Original targets | Baseline to 24 months | Model 1–3 | Analysis-specific cohorts | Table S6A |
| Alternative eGFR anchor | 3-month landmark | Does the 3-month target add prognostic information beyond kidney function at 3 months? | Composite target or UPCR criterion | 3 to 24 months | Includes 3-month eGFR | 3-month analytic cohort | Table S6B–C |
| Alternative eGFR anchor | 6-month landmark | Does the 6-month target add prognostic information beyond status at 6 months? | Original 6-month target | 6 to 24 months | Includes 6-month eGFR and UPCR | 6-month analytic cohort | Table S6D |
| Robustness | Model and selection assumptions | Are the primary estimates robust to alternative analytical assumptions? | Original targets | Primary outcome | Covariate substitutions, IPW, era, clustered SE | Analysis-specific cohorts | Tables S7, S8 |

| Analysis family | Analysis | Question addressed | Exposure | eGFR outcome anchor | Key model feature | Cohort | Reported in |
|---|---|---|---|---|---|---|---|
| Response timing | Timing of target achievement | Is earlier target achievement associated with the primary kidney outcome? | Target achieved by 3 months, first achieved at 6 months, or not achieved by 6 months | Primary outcome | Same covariates as the fully adjusted primary association model | Patients with evaluable 3- and 6-month response data | Figure 4 |

*Footnote.* The primary outcome was defined as a ≥30% decline in eGFR at 24 months from the higher of baseline or 3-month eGFR. EULAR, European League Against Rheumatism; UPCR, urine protein-to-creatinine ratio; eGFR, estimated glomerular filtration rate.

**Supplementary Table S2. Baseline characteristics and response/outcome availability of patients included in and excluded from the 3-month analytic cohort**

| Variable | Included in the 3-month analytic cohort | Excluded from the 3-month analytic cohort | SMD |
|---|---|---|---|
| n | 293 | 124 | |
| Age (years) | 40.3 ± 15.7 | 39.3 ± 13.8 | 0.065 |
| Female | 244 (83.3%) | 105 (84.7%) | 0.038 |
| BMI | 21.7 ± 3.5 | 22.3 ± 4.5 | 0.154 |
| SLEDAI | 20.6 ± 7.2 | 19.9 ± 6.6 | 0.100 |
| Laboratory data | | | |
| eGFR (ml/min/1.73m$^2$) | 75.4 ± 33.1 | 73.1 ± 33.8 | 0.067 |
| UPCR (g/g) | 3.3 ± 3.5 | 3.0 ± 3.0 | 0.096 |
| C3 (mg/dl) | 43.8 ± 22.9 | 44.6 ± 18.4 | 0.040 |
| C4 (mg/dl) | 7.2 ± 7.0 | 7.1 ± 6.0 | 0.022 |
| Histological findings | | | |
| Membranous mixed LN | 102 (34.8%) | 32 (25.8%) | 0.197 |
| Presence of chronic lesions | 142 (48.5%) | 58 (46.8%) | 0.034 |
| Treatment | | | |
| Use of HCQ | 113 (38.6%) | 56 (45.2%) | 0.134 |
| Intravenous GC pulse therapy | 152 (51.9%) | 65 (52.4%) | 0.011 |
| Initial prednisolone-equivalent dose (mg/kg/day) | 0.9 ± 0.2 | 0.9 ± 0.3 | 0.092 |
| Use of CY or MMF | 221 (75.4%) | 83 (66.9%) | 0.188 |
| Response and outcome assessment availability | | | |
| 3-month response assessment available | 293/293 (100.0%) | 31/124 (25.0%) | |
| 3-month target achiever among evaluable patients | 213/293 (72.7%) | 26/31 (83.9%) | 0.274 |
| 6-month response assessment available | 288/293 (98.3%) | 85/124 (68.5%) | |
| 6-month target achiever among evaluable patients | 246/288 (85.4%) | 72/85 (84.7%) | 0.020 |
| 24-month outcome available | 293/293 (100.0%) | 38/124 (30.6%) | |

*Footnote.* Data are presented as mean ± SD or n (%). The 3-month analytic cohort was defined by the availability of 3-month EULAR response status, baseline covariates, and the primary outcome. Standardized mean differences (SMDs) are shown to describe the magnitude of differences between groups. BMI, body mass index;

SLEDAI, Systemic Lupus Erythematosus Disease Activity Index; eGFR, estimated glomerular filtration rate; UPCR, urine protein-to-creatinine ratio; LN, lupus nephritis; GC, glucocorticoid; CY, cyclophosphamide; MMF, mycophenolate mofetil; HCQ, hydroxychloroquine; SMD, standardized mean difference.

**Supplementary Table S3. Crude longitudinal UPCR and eGFR values stratified by EULAR target achievement and occurrence of the primary kidney outcome at 24 months**

**Panel A. EULAR treatment target achievement at 3 and 6 months**

| **Variables** | **3-month target achiever** | **3-month target non-achiever** | **6-month target achiever** | **6-month target non-achiever** |
|---|---|---|---|---|
| **Number of patients** | 213 | 80 | 246 | 42 |
| **UPCR (g/g)** | | | | |
| Baseline (0 month) | 3.62 ± 3.52 | 2.58 ± 3.24 | 3.55 ± 3.59 | 1.93 ± 2.47 |
| 3 months | 0.87 ± 1.27 | 2.15 ± 3.12 | 1.00 ± 1.67 | 2.27 ± 3.19 |
| 6 months | 0.89 ± 5.77 | 0.90 ± 1.58 | 0.43 ± 0.78 | 3.64 ± 12.72 |
| 12 months | 0.32 ± 0.82 | 0.48 ± 0.88 | 0.27 ± 0.70 | 0.89 ± 1.30 |
| 24 months | 0.27 ± 0.70 | 0.37 ± 1.12 | 0.23 ± 0.59 | 0.71 ± 1.61 |
| **eGFR (ml/min/1.73m$^2$)** | | | | |
| Baseline (0 month) | 71.25 ± 30.40 | 86.33 ± 37.36 | 75.52 ± 32.16 | 73.67 ± 39.27 |
| 3 months | 82.95 ± 27.94 | 79.16 ± 30.59 | 82.19 ± 27.95 | 77.98 ± 33.43 |
| 6 months | 80.05 ± 26.34 | 76.31 ± 27.87 | 79.56 ± 26.17 | 73.84 ± 30.56 |
| 12 months | 78.54 ± 26.08 | 78.12 ± 28.41 | 79.30 ± 26.15 | 71.28 ± 29.71 |
| 24 months | 77.71 ± 26.26 | 77.92 ± 27.58 | 78.74 ± 26.29 | 69.47 ± 27.60 |

**Panel B. Occurrence of the primary kidney outcome at 24 months**

| Variables | Without the primary outcome at 24 months | With the primary outcome at 24 months |
|---|---|---|
| **Number of patients** | 251 | 42 |
| **UPCR (g/g)** | | |
| Baseline (0 month) | 3.22 ± 3.25 | 4.05 ± 4.57 |
| 3 months | 0.99 ± 1.54 | 2.62 ± 3.54 |
| 6 months | 0.78 ± 5.32 | 1.59 ± 2.20 |
| 12 months | 0.22 ± 0.37 | 1.17 ± 1.84 |
| 24 months | 0.19 ± 0.32 | 0.94 ± 1.91 |
| **eGFR (ml/min/1.73m$^2$)** | | |
| Baseline (0 month) | 76.17 ± 32.15 | 70.57 ± 38.23 |
| 3 months | 81.83 ± 27.35 | 82.38 ± 36.06 |
| 6 months | 80.49 ± 26.57 | 70.30 ± 26.66 |
| 12 months | 81.05 ± 26.09 | 62.72 ± 25.05 |
| 24 months | 81.62 ± 24.94 | 54.79 ± 24.64 |

*Footnote.* Data are presented as mean ± SD. Values are shown descriptively without adjustment. In Panel A, the 3- and 6-month analyses were performed in the 3-month and 6-month analytic cohorts, respectively. In Panel B, patients in the 3-month analytic cohort were stratified according to the occurrence of the primary outcome at 24 months. Number of patients indicates the number of patients in each group. Values at each time point are presented as mean ± SD among patients with available measurements. The primary outcome was defined as a ≥30% decline in eGFR at 24 months from the higher of baseline or 3-month eGFR. eGFR, estimated glomerular filtration rate; UPCR, urine protein-to-creatinine ratio.

**Supplementary Table S4. Full multivariable logistic regression models for a ≥30% decline in eGFR at 24 months according to achievement of the EULAR treatment targets at 3 and 6 months**

**Panel A: 3-month treatment response**

| | Model 1 | | | Model 2 | | | Model 3 | | |
|---|---|---|---|---|---|---|---|---|---|
| **Variable** | **OR** | **95% CI** | ***P* value** | **OR** | **95% CI** | ***P* value** | **OR** | **95% CI** | ***P* value** |
| Achievement of 3-month target | 0.42 | 0.21–0.84 | 0.015 | 0.39 | 0.19–0.80 | 0.010 | 0.40 | 0.20–0.82 | 0.013 |
| Male sex | 1.56 | 0.68–3.59 | 0.296 | 1.47 | 0.63–3.44 | 0.373 | 1.46 | 0.62–3.39 | 0.386 |
| Age (/ 10 year) | 1.26 | 1.02–1.54 | 0.029 | 1.25 | 0.98–1.58 | 0.067 | 1.22 | 0.96–1.55 | 0.100 |
| Baseline UPCR (per 1 g/g) | – | – | – | 1.07 | 0.98–1.17 | 0.135 | 1.08 | 0.98–1.18 | 0.115 |
| Baseline eGFR (per 10 ml/min/1.73m$^2$) | – | – | – | 1.00 | 0.89–1.13 | 0.979 | 1.01 | 0.89–1.14 | 0.909 |
| Presence of chronic lesions | – | – | – | – | – | – | 1.60 | 0.80–3.24 | 0.187 |

**Panel B: 6-month treatment response**

| | Model 1 | | | Model 2 | | | Model 3 | | |
|---|---|---|---|---|---|---|---|---|---|
| **Variable** | **OR** | **95% CI** | ***P* value** | **OR** | **95% CI** | ***P* value** | **OR** | **95% CI** | ***P* value** |
| Achievement of 6-month target | 0.45 | 0.20–1.00 | 0.049 | 0.38 | 0.17–0.87 | 0.023 | 0.40 | 0.17–0.91 | 0.030 |
| Male sex | 1.38 | 0.60–3.13 | 0.447 | 1.30 | 0.56–3.02 | 0.535 | 1.29 | 0.56–2.98 | 0.556 |
| Age (/ 10 year) | 1.23 | 1.01–1.51 | 0.044 | 1.26 | 0.99–1.59 | 0.058 | 1.23 | 0.97–1.57 | 0.092 |
| Baseline UPCR (per 1 g/g) | – | – | – | 1.09 | 0.99–1.19 | 0.077 | 1.09 | 0.99–1.19 | 0.066 |
| Baseline eGFR (per 10 ml/min/1.73m$^2$) | – | – | – | 1.04 | 0.92–1.17 | 0.560 | 1.04 | 0.92–1.17 | 0.527 |
| Presence of chronic lesions | – | – | – | – | – | – | 1.59 | 0.79–3.21 | 0.193 |

*Footnote.* Odds ratios were estimated using multivariable logistic regression models with a ≥30% decline in eGFR at 24 months from the higher of baseline or 3-month eGFR as the dependent variable. Separate models were fitted for the 3-month and 6-month response definitions. Model 1 was adjusted for sex and age; Model 2 was additionally adjusted for baseline UPCR and baseline eGFR; and Model 3 was further adjusted for the presence of chronic lesions. eGFR, estimated glomerular filtration rate; UPCR, urine protein-to-creatinine ratio; OR, odds ratio; CI, confidence interval.

**Supplementary Table S5. Full multivariable logistic regression models for the individual criteria of the 3-month EULAR target**

**Panel A. UPCR-reduction criterion entered separately**

| | Model 1 | | | Model 2 | | | Model 3 | | |
|---|---|---|---|---|---|---|---|---|---|
| **Variable** | **OR** | **95% CI** | ***P* value** | **OR** | **95% CI** | ***P* value** | **OR** | **95% CI** | ***P* value** |
| UPCR reduction ≥25% at 3 months | 0.47 | 0.21–1.05 | 0.066 | 0.42 | 0.18–0.96 | 0.040 | 0.45 | 0.19–1.04 | 0.060 |
| Male sex | 1.48 | 0.65–3.36 | 0.354 | 1.42 | 0.62–3.28 | 0.408 | 1.40 | 0.61–3.23 | 0.429 |
| Age (/ 10 year) | 1.28 | 1.04–1.56 | 0.020 | 1.32 | 1.04–1.67 | 0.023 | 1.29 | 1.01–1.64 | 0.040 |
| Baseline UPCR (per 1 g/g) | – | – | – | 1.07 | 0.98–1.18 | 0.116 | 1.08 | 0.98–1.18 | 0.103 |
| Baseline eGFR (per 10 ml/min/1.73m$^2$) | – | – | – | 1.04 | 0.92–1.18 | 0.486 | 1.05 | 0.93–1.18 | 0.463 |
| Presence of chronic lesions | – | – | – | – | – | – | 1.55 | 0.77–3.13 | 0.219 |

**Panel B. eGFR-preservation criterion entered separately**

| | Model 1 | | | Model 2 | | | Model 3 | | |
|---|---|---|---|---|---|---|---|---|---|
| **Variable** | **OR** | **95% CI** | ***P* value** | **OR** | **95% CI** | ***P* value** | **OR** | **95% CI** | ***P* value** |
| eGFR preservation ≥80% of baseline at 3 months | 0.51 | 0.22–1.18 | 0.116 | 0.47 | 0.18–1.21 | 0.117 | 0.45 | 0.17–1.17 | 0.100 |
| Male sex | 1.51 | 0.66–3.45 | 0.327 | 1.43 | 0.62–3.32 | 0.401 | 1.43 | 0.62–3.32 | 0.400 |
| Age (/ 10 year) | 1.25 | 1.02–1.53 | 0.033 | 1.23 | 0.96–1.57 | 0.097 | 1.19 | 0.93–1.53 | 0.169 |
| Baseline UPCR (per 1 g/g) | – | – | – | 1.06 | 0.97–1.16 | 0.209 | 1.06 | 0.97–1.16 | 0.185 |
| Baseline eGFR (per 10 ml/min/1.73m$^2$) | – | – | – | 0.99 | 0.87–1.13 | 0.907 | 0.99 | 0.87–1.14 | 0.917 |
| Presence of chronic lesions | – | – | – | – | – | – | 1.72 | 0.86–3.45 | 0.127 |

**Panel C. UPCR-reduction and eGFR-preservation criteria entered simultaneously**

| | Model 1 | | | Model 2 | | | Model 3 | | |
|---|---|---|---|---|---|---|---|---|---|
| Variable | OR | 95% CI | *P* value | OR | 95% CI | *P* value | OR | 95% CI | *P* value |
| UPCR reduction ≥25% at 3 months | 0.43 | 0.19–0.98 | 0.045 | 0.39 | 0.17–0.91 | 0.029 | 0.42 | 0.18–0.98 | 0.045 |
| eGFR preservation ≥80% of baseline at 3 months | 0.46 | 0.19–1.09 | 0.079 | 0.43 | 0.16–1.12 | 0.084 | 0.42 | 0.16–1.09 | 0.075 |
| Male sex | 1.58 | 0.68–3.63 | 0.285 | 1.49 | 0.63–3.48 | 0.361 | 1.47 | 0.63–3.44 | 0.371 |
| Age (/ 10 year) | 1.26 | 1.02–1.55 | 0.029 | 1.25 | 0.98–1.60 | 0.077 | 1.22 | 0.95–1.56 | 0.126 |
| Baseline UPCR (per 1 g/g) | – | – | – | 1.07 | 0.98–1.18 | 0.127 | 1.08 | 0.98–1.18 | 0.114 |
| Baseline eGFR (per 10 ml/min/1.73m$^2$) | – | – | – | 1.00 | 0.88–1.14 | 0.986 | 1.00 | 0.88–1.15 | 0.973 |
| Presence of chronic lesions | – | – | – | – | – | – | 1.60 | 0.79–3.23 | 0.195 |

*Footnote.* Odds ratios were estimated using multivariable logistic regression models with a ≥30% decline in eGFR at 24 months from the higher of baseline or 3-month eGFR as the dependent variable. In Panels A and B, the UPCR-reduction and eGFR-preservation criteria were entered separately into the models. In Panel C, both criteria were entered simultaneously into the models. Model 1 was adjusted for sex and age; Model 2 was additionally adjusted for baseline UPCR and baseline eGFR; and Model 3 was further adjusted for the presence of chronic lesions. EULAR, European League Against Rheumatism; eGFR, estimated glomerular filtration rate; UPCR, urine protein-to-creatinine ratio; OR, odds ratio; CI, confidence interval.

**Supplementary Table S6. Alternative eGFR-anchor analyses using baseline-referenced and landmark outcomes**

**Panel A. Analyses using an alternative outcome defined as a ≥30% decline in eGFR from baseline to 24 months**

**A-1. 3-month EULAR target**

| Variable | Model 1 | | | Model 2 | | | Model 3 | | |
|---|---|---|---|---|---|---|---|---|---|
| | OR | 95% CI | *P* value | OR | 95% CI | *P* value | OR | 95% CI | *P* value |
| Achievement of 3-month target | 0.27 | 0.10–0.74 | 0.010 | 0.43 | 0.15–1.25 | 0.121 | 0.43 | 0.15–1.26 | 0.126 |
| Male sex | 0.73 | 0.18–2.93 | 0.660 | 0.98 | 0.24–3.98 | 0.974 | 0.90 | 0.22–3.68 | 0.886 |
| Age (/ 10 year) | 1.51 | 1.12–2.04 | 0.007 | 2.05 | 1.35–3.09 | 0.001 | 2.05 | 1.34–3.14 | 0.001 |
| Baseline UPCR (per 1 g/g) | – | – | – | 0.93 | 0.76–1.13 | 0.473 | 0.95 | 0.78–1.15 | 0.599 |
| Baseline eGFR (per 10 ml/min/1.73m$^2$) | – | – | – | 1.29 | 1.04–1.60 | 0.021 | 1.32 | 1.06–1.65 | 0.015 |
| Presence of chronic lesions | – | – | – | – | – | – | 1.93 | 0.64–5.81 | 0.240 |

**A-2. 6-month EULAR target**

| Variable | Model 1 | | | Model 2 | | | Model 3 | | |
|---|---|---|---|---|---|---|---|---|---|
| | OR | 95% CI | *P* value | OR | 95% CI | *P* value | OR | 95% CI | *P* value |
| Achievement of 6-month target | 0.35 | 0.12–1.01 | 0.051 | 0.44 | 0.14–1.41 | 0.168 | 0.48 | 0.15–1.54 | 0.215 |
| Male sex | 0.64 | 0.17–2.48 | 0.520 | 0.91 | 0.22–3.66 | 0.891 | 0.83 | 0.20–3.39 | 0.796 |
| Age (/ 10 year) | 1.47 | 1.09–1.99 | 0.012 | 2.07 | 1.38–3.13 | <0.001 | 2.07 | 1.35–3.15 | 0.001 |
| Baseline UPCR (per 1 g/g) | – | – | – | 0.94 | 0.77–1.15 | 0.548 | 0.95 | 0.78–1.16 | 0.639 |
| Baseline eGFR (per 10 ml/min/1.73m$^2$) | – | – | – | 1.33 | 1.09–1.64 | 0.006 | 1.35 | 1.10–1.67 | 0.005 |
| Presence of chronic lesions | – | – | – | – | – | – | 1.83 | 0.61–5.52 | 0.282 |

**Panel B. Three-month landmark analysis of the 3-month EULAR target**

| Variable | Model 1 | | | Model 2 | | | Model 3 | | |
|---|---|---|---|---|---|---|---|---|---|
| | OR | 95% CI | *P* value | OR | 95% CI | *P* value | OR | 95% CI | *P* value |
| Achievement of 3-month target | 0.90 | 0.39–2.05 | 0.793 | 0.73 | 0.31–1.71 | 0.464 | 0.75 | 0.31–1.76 | 0.504 |
| Male sex | 1.44 | 0.57–3.63 | 0.443 | 1.35 | 0.51–3.58 | 0.544 | 1.31 | 0.49–3.49 | 0.586 |
| Age (/ 10 year) | 1.10 | 0.87–1.39 | 0.409 | 1.36 | 1.00–1.86 | 0.050 | 1.33 | 0.97–1.83 | 0.074 |
| Baseline UPCR (per 1 g/g) | – | – | – | 1.13 | 1.03–1.23 | 0.010 | 1.13 | 1.03–1.24 | 0.008 |
| 3-month eGFR (per 10 ml/min/1.73m$^2$) | – | – | – | 1.22 | 1.04–1.44 | 0.013 | 1.24 | 1.05–1.45 | 0.011 |
| Presence of chronic lesions | – | – | – | – | – | – | 1.86 | 0.83–4.17 | 0.129 |

**Panel C. Three-month landmark analysis of the UPCR-reduction criterion**

| Variable | Model 1 | | | Model 2 | | | Model 3 | | |
|---|---|---|---|---|---|---|---|---|---|
| | OR | 95% CI | *P* value | OR | 95% CI | *P* value | OR | 95% CI | *P* value |
| UPCR reduction ≥25% at 3 months | 0.36 | 0.15–0.86 | 0.021 | 0.31 | 0.13–0.78 | 0.013 | 0.34 | 0.14–0.86 | 0.022 |
| Male sex | 1.49 | 0.59–3.79 | 0.402 | 1.35 | 0.51–3.62 | 0.545 | 1.31 | 0.49–3.52 | 0.589 |
| Age (/ 10 year) | 1.11 | 0.88–1.41 | 0.369 | 1.36 | 0.99–1.87 | 0.055 | 1.34 | 0.97–1.84 | 0.075 |
| Baseline UPCR (per 1 g/g) | – | – | – | 1.14 | 1.04–1.25 | 0.005 | 1.14 | 1.04–1.25 | 0.004 |
| 3-month eGFR (per 10 ml/min/1.73m$^2$) | – | – | – | 1.21 | 1.03–1.41 | 0.021 | 1.21 | 1.03–1.42 | 0.017 |
| Presence of chronic lesions | – | – | – | – | – | – | 1.69 | 0.74–3.83 | 0.211 |

**Panel D. Six-month landmark analysis of the 6-month EULAR target**

| Model | Covariates | OR for 6-month EULAR target achievement | 95% CI | P value |
|---|---|---|---|---|
| Landmark Model 1 | age, sex | 0.85 | 0.23–3.13 | 0.804 |
| Landmark Model 2 | Model 1 + 6-month eGFR | 0.86 | 0.23–3.19 | 0.822 |
| Landmark Model 3 | Model 2 + 6-month UPCR | 1.00 | 0.25–4.06 | 0.999 |

*Footnote.* In Panel A, the outcome was defined as a ≥30% decline in eGFR from baseline to 24 months. In Panels B and C, the outcome was defined as a ≥30% decline in eGFR from 3 to 24 months. In Panel D, the outcome was defined as a ≥30% decline in eGFR from 6 to 24 months. Odds ratios were estimated using multivariable logistic regression models. In Panel A, Model 1 was adjusted for sex and age; Model 2 was additionally adjusted for baseline UPCR and baseline eGFR; and Model 3 was further adjusted for the presence of chronic lesions. In Panels B and C, Model 1 was adjusted for sex and age; Model 2 was additionally adjusted for baseline UPCR and 3-month eGFR; and Model 3 was further adjusted for the presence of chronic lesions. In Panel D, Model 1 was adjusted for sex and age; Model 2 was additionally adjusted for 6-month eGFR; and Model 3 was further adjusted for 6-month UPCR. The 3- and 6-month analyses were performed in the 3-month analytic cohort (n = 293) and the 6-month analytic cohort (n = 288), respectively. The number of outcome events was 42 for the primary outcome (≥30% decline in eGFR from the higher of baseline or 3-month eGFR), 18 in Panel A (in both A-1 and A-2), 31 in Panels B and C, and 17 in Panel D. EULAR, European League Against Rheumatism; eGFR, estimated glomerular filtration rate; UPCR, urine protein-to-creatinine ratio; OR, odds ratio; CI, confidence interval.

**Supplementary Table S7. Robustness analyses of multivariable logistic regression models replacing chronic lesions with either intravenous glucocorticoid pulse therapy during initial treatment or mixed proliferative/membranous histology (class III/IV + V)**

**Panel A: 3-month treatment response**

| Variable | Intravenous GC pulse therapy instead of chronic lesions | | | Mixed proliferative/membranous histology instead of chronic lesions | | |
|---|---|---|---|---|---|---|
| | **OR** | **95% CI** | ***P* value** | **OR** | **95% CI** | ***P* value** |
| Achievement of 3-month target | 0.39 | 0.19–0.80 | 0.010 | 0.39 | 0.19–0.80 | 0.010 |
| Male sex | 1.48 | 0.63–3.48 | 0.364 | 1.47 | 0.63–3.45 | 0.373 |
| Age (/ 10 year) | 1.24 | 0.98–1.57 | 0.077 | 1.25 | 0.99–1.58 | 0.066 |
| Baseline UPCR (per 1 g/g) | 1.07 | 0.98–1.18 | 0.126 | 1.07 | 0.98–1.18 | 0.134 |
| Baseline eGFR (per 10 ml/min/1.73m$^2$) | 1.00 | 0.88–1.13 | 0.962 | 1.00 | 0.89–1.14 | 0.953 |
| Intravenous GC pulse therapy | 0.87 | 0.43–1.76 | 0.706 | – | – | – |
| Mixed proliferative/membranous histology (class III/IV + V) | – | – | – | 0.93 | 0.45–1.92 | 0.853 |

**Panel B: 6-month treatment response**

| Variable | Intravenous GC pulse therapy instead of chronic lesions | | | Mixed proliferative/membranous histology instead of chronic lesions | | |
|---|---|---|---|---|---|---|
| | **OR** | **95% CI** | ***P* value** | **OR** | **95% CI** | ***P* value** |
| Achievement of 6-month target | 0.38 | 0.17–0.87 | 0.022 | 0.37 | 0.16–0.86 | 0.021 |
| Male sex | 1.33 | 0.57–3.08 | 0.512 | 1.31 | 0.56–3.03 | 0.534 |
| Age (/ 10 year) | 1.25 | 0.98–1.59 | 0.067 | 1.26 | 0.99–1.60 | 0.055 |
| Baseline UPCR (per 1 g/g) | 1.09 | 0.99–1.19 | 0.069 | 1.09 | 0.99–1.19 | 0.070 |
| Baseline eGFR (per 10 ml/min/1.73m$^2$) | 1.03 | 0.91–1.16 | 0.631 | 1.04 | 0.92–1.18 | 0.517 |
| Intravenous GC pulse therapy | 0.85 | 0.42–1.70 | 0.639 | – | – | – |
| Mixed proliferative/membranous histology (class III/IV + V) | – | – | – | 0.87 | 0.42–1.79 | 0.700 |

*Footnote.* Odds ratios were estimated using the fully adjusted multivariable logistic regression model with a ≥30% decline in eGFR at 24 months from the higher of baseline or 3-month eGFR as the dependent variable. Separate models were fitted for the 3-month and 6-month response definitions. The model was adjusted for sex,

age, baseline UPCR, baseline eGFR, and either intravenous GC pulse therapy during the initial treatment phase or mixed proliferative/membranous histology (class III/IV + V), in place of the presence of chronic lesions. eGFR, estimated glomerular filtration rate; UPCR, urine protein-to-creatinine ratio; OR, odds ratio; CI, confidence interval; GC, glucocorticoid.

**Supplementary Table S8. Robustness analyses using inverse probability weighting, treatment-era adjustment, and center-clustered robust standard errors**

**Panel A. Inverse probability weighting analysis**

| Exposure | Analysis | Adjusted OR | 95% CI | P value |
|---|---|---|---|---|
| 3-month EULAR target | | | | |
| | Primary complete-case analysis | 0.40 | 0.20–0.82 | 0.013 |
| | IPW-weighted analysis | 0.37 | 0.18–0.79 | 0.010 |
| 6-month EULAR target | | | | |
| | Primary complete-case analysis | 0.40 | 0.17–0.91 | 0.030 |
| | IPW-weighted analysis | 0.38 | 0.16–0.90 | 0.027 |

**Panel B. Additional adjustment for treatment era**

| | Primary Model 3 | | | Model 3 + treatment era | | |
|---|---|---|---|---|---|---|
| **Response definition** | **OR** | **95% CI** | **P value** | **OR** | **95% CI** | **P value** |
| 3-month EULAR target | 0.40 | 0.20–0.82 | 0.013 | 0.39 | 0.19–0.81 | 0.012 |
| 6-month EULAR target | 0.40 | 0.17–0.91 | 0.030 | 0.43 | 0.18–0.99 | 0.047 |

**Panel C. Analysis using center-clustered robust standard errors**

| | Primary Model 3 | | | Center-clustered robust Model 3 | | |
|---|---|---|---|---|---|---|
| **Response definition** | **OR** | **95% CI** | **P value** | **OR** | **95% CI** | **P value** |
| 3-month EULAR target | 0.40 | 0.20–0.82 | 0.013 | 0.40 | 0.17–0.94 | 0.035 |
| 6-month EULAR target | 0.40 | 0.17–0.91 | 0.030 | 0.40 | 0.16–1.00 | 0.051 |

*Footnote.* Odds ratios were estimated using multivariable logistic regression models. Primary Model 3 was adjusted for sex, age, baseline UPCR, baseline eGFR, and the presence of chronic lesions. In Panel A, stabilized inverse probability weights for inclusion in each analysis-specific complete-case cohort were estimated among adult

patients with available baseline variables using age, sex, baseline UPCR, baseline eGFR, the presence of chronic lesions, and treatment era. The weights were truncated at the 1st and 99th percentiles and applied to Model 3. After weighting, all absolute standardized mean differences between the weighted complete-case cohort and the corresponding source cohort with complete IPW-model covariates (n = 389) were <0.01. In Panel B, treatment era, categorized according to the year of treatment initiation as ≤2009, 2010–2016, and ≥2017, was additionally included in Model 3. In Panel C, center-clustered robust standard errors were estimated for Model 3. The 3- and 6-month analyses were performed in the 3-month analytic cohort (n = 293) and the 6-month analytic cohort (n = 288), respectively. EULAR, European League Against Rheumatism; eGFR, estimated glomerular filtration rate; UPCR, urine protein-to-creatinine ratio; IPW, inverse probability weighting; OR, odds ratio; CI, confidence interval.

**Supplementary Table S9. Sensitivity analysis of between-group differences in eGFR change from 3 months onward using a modified 3-month target definition based on the UPCR criterion alone**

| Visit | Adjusted difference in change in eGFR from 3 months onward between achievers and non-achievers (ml/min/1.73m²) | 95% CI | *P* value |
|---|---|---|---|
| 6 months | 8.21 | 2.92–13.50 | 0.002 |
| 12 months | 6.30 | 1.01–11.59 | 0.020 |
| 24 months | 6.04 | 0.75–11.33 | 0.025 |

*Footnote.* Values represent adjusted between-group differences in change in eGFR from 3 months onward between achievers and non-achievers. Positive β values indicate that achievers showed a more favorable change in eGFR after 3 months (i.e., a greater increase or a smaller decline) than non-achievers. In this sensitivity analysis, the 3-month target was redefined by removing the eGFR component from the original EULAR definition and retaining only the UPCR criterion. Estimates were obtained from linear mixed-effects models adjusted for sex, age, baseline UPCR, baseline eGFR, and the presence of chronic lesions. CI, confidence interval; eGFR, estimated glomerular filtration rate; UPCR, urine protein-to-creatinine ratio.

STROBE Statement—Checklist of items that should be included in reports of ***cohort studies***

| | Item No | Recommendation | Comment |
|---|---|---|---|
| **Title and abstract** | 1 | (*a*) Indicate the study's design with a commonly used term in the title or the abstract | The study design is indicated in the abstract as a multicenter retrospective cohort study. |
| | | (*b*) Provide in the abstract an informative and balanced summary of what was done and what was found | The abstract summarizes background, methods, results, and conclusions with key numerical findings. |
| **Introduction** | | | |
| Background/rationale | 2 | Explain the scientific background and rationale for the investigation being reported | The Introduction explains the clinical importance of early treatment targets in lupus nephritis and the limited evidence supporting the prognostic value of the EULAR 3- and 6-month targets. |
| Objectives | 3 | State specific objectives, including any prespecified hypotheses | The final paragraph of the Introduction states the study objective: to examine whether achievement of the EULAR-recommended treatment response at 3 and 6 months is associated with a ≥30% decline in eGFR at 24 months. |
| **Methods** | | | |
| Study design | 4 | Present key elements of study design early in the paper | Key elements of the study design are presented at the beginning of the Methods section as a multicenter, retrospective cohort analysis. |
| Setting | 5 | Describe the setting, locations, and relevant dates, including periods of recruitment, exposure, follow-up, and data collection | The Methods describe the multicenter setting, eligibility period, and timing of baseline and follow-up data collection, including assessments at approximately 3, 6, 12, and 24 months after treatment initiation. |
| Participants | 6 | (*a*) Give the eligibility criteria, and the sources and methods of selection of participants. Describe methods of follow-up | Eligibility criteria, diagnostic criteria, sources of participants, and cohort construction are described in the Methods, and patient selection is illustrated in Figure 1. |
| | | (*b*) For matched studies, give matching criteria and number of exposed and unexposed | Not applicable. |
| Variables | 7 | Clearly define all outcomes, exposures, predictors, potential confounders, and effect | The exposure, outcome, covariates, and sensitivity-analysis definitions are clearly described in the Methods, including the EULAR 3- and 6-month |

| | | | |
|---|---|---|---|
| | | modifiers. Give diagnostic criteria, if applicable | treatment targets and the primary kidney outcome. |
| Data sources/ measurement | 8* | For each variable of interest, give sources of data and details of methods of assessment (measurement). Describe comparability of assessment methods if there is more than one group | Data sources and measurement methods are described in the Data collection section, including baseline biopsy-based variables, laboratory assessments, treatment information, and prespecified visit windows. |
| Bias | 9 | Describe any efforts to address potential sources of bias | Potential sources of bias were addressed through multivariable adjustment and sensitivity analyses. Residual confounding and possible misclassification are discussed as limitations. |
| Study size | 10 | Explain how the study size was arrived at | The study size was determined by the number of eligible patients available in the participating centers during the study period; no formal sample-size calculation was performed for this retrospective cohort study. |
| Quantitative variables | 11 | Explain how quantitative variables were handled in the analyses. If applicable, describe which groupings were chosen and why | Quantitative variables were analyzed as continuous variables where appropriate, and treatment response was categorized according to the predefined EULAR target thresholds at 3 and 6 months. |
| Statistical methods | 12 | (*a*) Describe all statistical methods, including those used to control for confounding | Statistical methods are described in the Statistical Analysis section, including multivariable logistic regression for the primary outcome and linear mixed-effects models for longitudinal eGFR trajectories. |
| | | (*b*) Describe any methods used to examine subgroups and interactions | Additional analyses included transition-based logistic regression according to timing of target achievement and sensitivity analyses using alternative model specifications and response definitions. |
| | | (*c*) Explain how missing data were addressed | Complete-case analysis was used for the primary models, with analysis-specific cohorts defined according to the availability of the required baseline, exposure, and outcome variables. To assess potential selection bias due to missing data, a robustness analysis was performed using stabilized inverse probability weights for inclusion in each complete-case cohort, with weights |

| | | | |
|---|---|---|---|
| | | | truncated at the 1st and 99th percentiles. |
| | | (*d*) If applicable, explain how loss to follow-up was addressed | Because this was a retrospective cohort with outcome assessment at prespecified time windows, loss to follow-up was handled by restricting each analysis to patients with evaluable data for the relevant response and outcome variables. |
| | | (*e*) Describe any sensitivity analyses | Additional analyses included component analyses of the 3-month target; alternative eGFR-anchor analyses using a baseline-referenced outcome and 3- and 6-month landmark outcomes; robustness analyses using alternative covariate specifications, inverse probability weighting, treatment-era adjustment, and center-clustered robust standard errors; and a sensitivity analysis using the UPCR criterion alone. |
| **Results** | | | |
| Participants | 13* | (a) Report numbers of individuals at each stage of study—eg numbers potentially eligible, examined for eligibility, confirmed eligible, included in the study, completing follow-up, and analysed | The numbers of patients at each stage of study inclusion are reported in the Results and shown in Figure 1, from 449 initially identified patients to the final analytic cohorts. |
| | | (b) Give reasons for non-participation at each stage | Reasons for exclusion, including age <18 years and missing data required for inclusion in the 3-month analytic cohort, are reported in the Results and detailed in Figure 1. |
| | | (c) Consider use of a flow diagram | A participant flow diagram is provided as Figure 1. |
| Descriptive data | 14* | (a) Give characteristics of study participants (eg demographic, clinical, social) and information on exposures and potential confounders | Baseline demographic, clinical, histological, and treatment characteristics of the analytic cohorts are presented in Table 1, and characteristics of included and excluded patients are shown in Supplementary Table S2. |
| | | (b) Indicate number of participants with missing data for each variable of interest | The analytic cohort was defined according to the availability of key variables required for the main analyses, and exclusions due to unavailable follow-up data are |

| | | | |
|---|---|---|---|
| | | | described in the Methods and flow diagram. |
| | | (c) Summarise follow-up time (eg, average and total amount) | The timing of outcome assessment and longitudinal follow-up is described using prespecified visits at approximately 3, 6, 12, and 24 months after treatment initiation. |
| Outcome data | 15* | Report numbers of outcome events or summary measures over time | Numbers of outcome events are reported in Table 2, and longitudinal outcome patterns are additionally shown in Figure 2, Figure 3, Figure 4, and the Supplementary Materials. |
| Main results | 16 | (*a*) Give unadjusted estimates and, if applicable, confounder-adjusted estimates and their precision (eg, 95% confidence interval). Make clear which confounders were adjusted for and why they were included | Confounder-adjusted estimates with 95% confidence intervals are presented in Table 2 and Supplementary Table S4, with covariates prespecified in sequential models and explicitly described in the Methods. |
| | | (*b*) Report category boundaries when continuous variables were categorized | Category boundaries for treatment response were prespecified according to the EULAR definitions: a ≥25% reduction in UPCR with preserved kidney function at 3 months and a ≥50% reduction in UPCR at 6 months. |
| | | (*c*) If relevant, consider translating estimates of relative risk into absolute risk for a meaningful time period | Absolute risks are presented as observed event proportions in Table 2, and model-based adjusted probabilities are described in the Results section. |
| Other analyses | 17 | Report other analyses done—eg analyses of subgroups and interactions, and sensitivity analyses | Other analyses included longitudinal mixed-effects modeling of eGFR trajectories, Sankey visualization of response transitions, transition-based multivariable logistic regression, and sensitivity analyses reported in the Supplementary Materials. |
| **Discussion** | | | |
| Key results | 18 | Summarise key results with reference to study objectives | The first paragraph of the Discussion summarizes the main findings in relation to the study objective. |
| Limitations | 19 | Discuss limitations of the study, taking into account sources of potential bias or imprecision. Discuss both direction and magnitude of any potential bias | The Discussion addresses important limitations, including the retrospective observational design, possible residual confounding, temporal changes in clinical practice, potential misclassification due to visit windows, |

| | | | |
|---|---|---|---|
| | | | and the limited assessment of longer-term prognosis. |
| Interpretation | 20 | Give a cautious overall interpretation of results considering objectives, limitations, multiplicity of analyses, results from similar studies, and other relevant evidence | The Discussion provides a cautious interpretation of the findings in the context of previous studies, the predefined study objective, the sensitivity analyses, and the study limitations. |
| Generalisability | 21 | Discuss the generalisability (external validity) of the study results | The generalizability of the findings is discussed in relation to the multicenter design, biopsy-proven proliferative lupus nephritis population, and real-world clinical practice. |
| **Other information** | | | |
| Funding | 22 | Give the source of funding and the role of the funders for the present study and, if applicable, for the original study on which the present article is based | The source of funding is reported in the Funding section. |

*Give information separately for exposed and unexposed groups.

**Note:** An Explanation and Elaboration article discusses each checklist item and gives methodological background and published examples of transparent reporting. The STROBE checklist is best used in conjunction with this article (freely available on the Web sites of PLoS Medicine at http://www.plosmedicine.org/, Annals of Internal Medicine at http://www.annals.org/, and Epidemiology at http://www.epidem.com/). Information on the STROBE Initiative is available at http://www.strobe-statement.org.
